# Mechanically aligned total knee arthroplasty demonstrates acceptable outcomes in obese patients, with insufficient evidence for alternative alignment strategies: A systematic review

**DOI:** 10.1002/jeo2.70860

**Published:** 2026-07-30

**Authors:** Helena Son, Andjela Dragovic, Marc Daniel Bouchard, Maaya Chander, Prushoth Vivekanantha, Hassaan Abdel Khalik, Naser Ali Alenezi, Luca Farinelli, Darren de SA, Amit Meena

**Affiliations:** ^1^ Michael DeGroote School of Medicine McMaster University Hamilton Ontario Canada; ^2^ Department of Molecular Biology University of Guelph Guelph Ontario Canada; ^3^ Division of Orthopaedic Surgery, Department of Surgery McMaster University Hamilton Ontario Canada; ^4^ Ron Joyce Children's Health Centre Dr. Lankesh Charity Foundation Hamilton Ontario Canada; ^5^ Clinical Orthopedics, Department of Clinical and Molecular Sciences Università Politecnica delle Marche Ancona Italy; ^6^ KNEECARES – The Superspeciality Knee Clinic Jaipur India; ^7^ Division of Orthopaedic Surgery Queen's University Kingston Ontario Canada

**Keywords:** kinematic alignment (KA), mechanical alignment (MA), obesity, total knee arthroplasty (TKA)

## Abstract

**Purpose:**

Obesity (body mass index [BMI] ≥ 30 kg/m^2^) is increasingly prevalent among total knee arthroplasty (TKA) patients and is associated with unique challenges. The role of various TKA alignment strategies in obese patients remains unclear. To evaluate the clinical, functional, radiographic, survivorship and complication outcomes of primary TKA in obese patients across alignment philosophies.

**Methods:**

Eligible studies (1) included patients who underwent primary TKA with defined alignment, (2) reported BMI ≥ 30 kg/m^2^ or provided stratified data for obese versus non‐obese groups, (3) reported at least one of: patient‐reported outcome measures (PROMs), range of motion (ROM), radiographic outcomes, or complications and (4) were randomized controlled trials (RCTs), cohort studies or case–control studies.

**Results:**

Thirty studies were included. 8468 obese patients (14,662 knees) underwent mechanically aligned TKA, with mean follow‐up of 91.1 ± 45.3 months (range, 0.5–110.5 months). Mechanically aligned TKA in obese patients was associated with post‐operative improvements in PROMs, including Knee Society Score, Western Ontario and McMaster Universities Osteoarthritis Index and Oxford Knee Score. Short‐term cumulative aseptic revision incidence was low, ranging from 0.5% to 1.3%, while revision‐free survivorship commonly exceeded 95% at short‐term and >90% at long‐term follow‐up. Radiographic outcomes demonstrated reliable restoration of near‐neutral limb alignment across BMI strata. One study evaluated a kinematically‐designed bicruciate‐stabilized implant with an unspecified alignment strategy in obese patients, and another study evaluated functional alignment in obese patients, limiting comparisons.

**Conclusion:**

Evidence evaluating the influence of alignment on outcomes in obese patients undergoing TKA remains insufficient to determine whether alignment philosophy meaningfully affects clinical or radiographic outcomes. Although mechanically aligned TKA demonstrated generally acceptable post‐operative outcomes in obese cohorts, the available literature was overwhelmingly dominated by mechanically aligned studies, with only limited evaluation of alternative approaches.

**Level of Evidence:**

Level IV.

## Graphical abstract

Systematic review evaluating clinical, radiographic, and survivorship outcomes of total knee arthroplasty (TKA) in obese patients according to alignment strategy. Mechanically‐aligned TKA demonstrated substantial postoperative improvements in patient‐reported outcome measures (PROMs), frequent achievement of Patient Acceptable Symptom State (PASS) thresholds, and acceptable short‐ and long‐term survivorship. Evidence for alternative alignment strategies was limited.

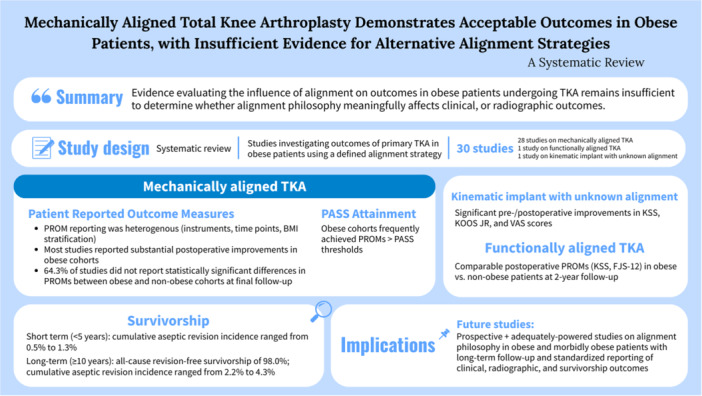

**Abbreviations**
BMI: body mass index
CIs: confidence intervals
FJS‐12: Forgotten Joint Score‐12
HKA: hip–knee–ankle
HSS: Hospital for Special Surgery Knee‐Rating Scale
ICC: intraclass correlation coefficient
IQRs: interquartile ranges
KA: kinematic alignment
KOOS: Knee Osteoarthritis and Outcome Score
KOOS‐JR: Knee Osteoarthritis and Outcome Score Joint Replacement
KSS: Knee Society Score
MA: mechanical alignment
MINORS: Methodological Index for Non‐Randomized Studies
mMPTA: mechanical medial proximal tibial angle
OKS: Oxford Knee Score
PASS: patient acceptable symptom state
PRISMA: Preferred Reporting Items for Systematic Reviews and Meta‐Analyses
PROMs: patient‐reported outcome measures
RCTs: randomized controlled trials
ROB2: risk of bias 2
ROC: receiver operating characteristic
ROM: range of motion
SDs: standard deviations
SF‐12: Short‐Form Questionnaire
TKA: total knee arthroplasty
WOMAC: Western Ontario and McMaster Universities Osteoarthritis Index

## INTRODUCTION

Total knee arthroplasty (TKA) is a highly successful intervention for end‐stage knee osteoarthritis, offering substantial pain relief and functional improvement [28, 75, 77]. However, the optimal alignment strategy during TKA remains debated, particularly in patients with obesity, where anatomical variability, soft‐tissue tensioning, and increased joint loads complicate surgical decision‐making [50]. Traditionally, mechanical alignment (MA), which involves restoring a neutral mechanical axis through standardized bone cuts, has been considered the gold standard for ensuring even load distribution and implant longevity [26, 80]. More recently, patient‐specific alignment philosophies, including but not limited to kinematic (KA), functional alignment (FA) and anatomical alignment (AA), have sought to restore the patient's native joint line and kinematics, potentially improving functional outcomes and satisfaction [20, 36, 41, 44, 80].

Obesity, defined as a body mass index (BMI) ≥ 30 kg/m^2^, presents unique biomechanical and clinical challenges in TKA [13, 53, 59, 60]. Increased body weight elevates joint reaction forces and may alter gait mechanics, contributing to greater component stresses, accelerated wear and higher complication rates [1, 40, 50]. Additionally, excessive soft‐tissue thickness and limb deformity can affect intraoperative exposure and alignment accuracy [21, 40, 50]. While multiple studies have assessed the influence of obesity on implant survival and post‐operative function, few have examined how alignment strategy interacts with BMI to influence outcomes. Given that both obesity and malalignment are independent predictors of implant failure, understanding their interplay is essential for optimizing TKA performance in this growing patient population.

Emerging evidence suggests that kinematic and other patient‐specific alignment approaches may improve early function and patient satisfaction in selected TKA populations [23, 35, 62]; however, evidence evaluating these approaches in obese patients remains limited and incompletely characterized. Obesity introduces unique technical and biomechanical considerations, including altered soft‐tissue tensioning, increased joint loads and challenges in surgical exposure, which may influence the performance and reproducibility of different alignment strategies. Some reports indicate that soft‐tissue balancing and ligament tensioning in obese knees may be less predictable under KA principles, potentially predisposing to instability or early wear; conversely, others propose that restoring constitutional alignment may mitigate excessive medial loading and better accommodate soft‐tissue envelope constraints in obesity [23, 37, 46, 57]. Although MA remains the predominant philosophy used in obese patients, it is unclear whether outcomes differ meaningfully across alignment approaches or whether alternative strategies can be safely and effectively applied in this higher‐risk population.

This systematic review aims to evaluate the clinical, functional, radiographic, survivorship and complication outcomes of primary TKA in obese patients across various alignment philosophies. The secondary aim of this study was to explore outcomes across obesity severity, stratifying patients by BMI categories (<30, 30–40 and >40 kg/m^2^) where reported, to assess whether alignment‐related outcomes may vary according to obesity class and whether alignment choice may warrant tailoring to obesity severity. It is hypothesized that existing literature on obese TKA would be predominantly based on MA, with limited comparative evidence to draw definitive conclusions regarding the relative performance of alternative alignment philosophies.

## METHODS

### Search strategy

A comprehensive literature search was conducted in accordance with the Preferred Reporting Items for Systematic Reviews and Meta‐Analyses (PRISMA) [55] guidelines across MEDLINE, EMBASE and Emcare databases from inception to 22 September 2025. The search strategy included terms related to ‘obesity’, ‘total knee arthroplasty’, ‘alignment’ and ‘body mass index’ in combination with Boolean operators. References of included studies and relevant systematic reviews were also manually screened for eligible articles. The full search strategy is available in Appendix S1.

Studies were eligible for inclusion if they: (1) included patients who underwent primary TKA with defined alignment (MA, KA, restricted kinematic [rKA], FA, AA, etc.), (2) reported BMI ≥ 30 kg/m^2^ (obese patients) or provided stratified data for obese versus non‐obese groups if the study population included mixed populations, (3) reported at least one of: clinical/functional outcomes (patient‐reported outcome measures [PROMs], pain, functional scores, range of motion [ROM]), radiographic outcomes or complications and (4) were randomized controlled trials (RCTs), prospective/retrospective cohort studies or case–control studies. Studies were excluded if they: (1) included patients who underwent revision TKA, unicompartmental TKA, or TKA following a failed procedure (e.g., high tibial osteotomy), (2) were reviews, meta‐analyses, level V studies, expert commentaries, technical notes, case reports/series with less than five patients, abstracts or ongoing clinical trials with no results available, (3) were not available in English or have an English translation available and (4) were non‐human studies (animal studies and cadaveric studies).

### Study screening

Two reviewers (H.S. and A.D.) independently reviewed titles and abstracts for eligibility in the Covidence online software (Veritas Health Innovation). Conflicts at this stage were automatically pushed towards full‐text screening to ensure that all relevant texts were included in the final search. Full texts of potentially eligible studies were retrieved and evaluated. Conflicts between authors were resolved through discussion and consensus. Discrepancies that could not be resolved between the two authors were resolved by a more senior author (M.D.B.). The reference lists of all included studies were also screened to ensure that no additional eligible articles meeting the inclusion criteria were overlooked.

### Data abstraction

Data were extracted by two reviewers (H.S. and A.D.) using a standardized data collection form in Google Sheets (Google LLC). Extracted data included study characteristics, demographic variables, surgical characteristics, PROMs at final follow‐up (e.g., Forgotten Joint Score‐12 [FJS‐12], Knee Osteoarthritis and Outcome Score [KOOS] and Western Ontario and McMaster Universities Osteoarthritis Index [WOMAC]), functional outcomes, survivorship rates, complications and radiographic outcomes (e.g., hip–knee–ankle [HKA] angle, femorotibial angle and post‐operative alignment). Discrepancies between reviewers during data extraction were resolved by discussion and consensus or through consultation with a more senior author (M.D.B.).

### Quality assessment

The methodological quality of included studies was assessed and scored using the Methodological Index for Non‐Randomized Studies (MINORS) criteria [74]. Two reviewers (H.S. and A.D.) independently scored each study across the 12 MINORS items, which assess various methodological domains. Discrepancies were resolved with discussion, and the intraclass correlation coefficient (ICC) was used to assess inter‐rater agreement. The risk of bias for the three included RCTs was evaluated using the risk of bias 2 (ROB2) tool [34].

### Alignment strategy classification

Studies were classified according to the alignment philosophy explicitly described by the authors of the original study. Alignment strategies were categorized as MA, KA, FA, rKA, AA or other patient‐specific alignment approaches where clearly defined. MA was defined as techniques aiming to restore a neutral mechanical axis (typically targeting approximately 180° HKA alignment or equivalent neutral coronal alignment) using standardized bone resections and ligament balancing. KA was defined as approaches seeking to restore the patient's native or constitutional joint line and limb alignment through individualized bone resections. FA and rKA were classified according to study‐specific definitions, typically incorporating patient‐specific constitutional alignment targets within predefined coronal or sagittal boundaries.

Importantly, surgical technologies or execution tools, including computer navigation, robotic assistance, accelerometer‐based guidance and patient‐specific instrumentation (PSI), were not independently classified as alignment philosophies, as these approaches may be used to execute different alignment strategies. Similarly, gap‐balancing techniques, tibial referencing methods or mild residual varus targets were not assumed to represent distinct alignment philosophies unless explicitly framed as such by study authors. Implant design alone was not considered sufficient to classify a study as KA. Studies evaluating kinematically designed, bicruciate‐stabilized, guided‐motion or other implant systems were classified according to the reported alignment target. Studies describing navigation‐assisted or robotic TKA targeting neutral alignment were classified as MA, whereas studies explicitly restoring constitutional alignment or patient‐specific coronal targets were categorized according to the stated philosophy. In cases of ambiguity, classification was determined by reviewer consensus based on the stated alignment target, coronal alignment goals and surgical principles described in the study methodology. When no alignment philosophy or bone‐resection target was described, the alignment strategy was classified as unspecified.

### Patient acceptable symptom state (PASS) thresholds

Where scale directionality and reporting format permitted, post‐operative PROMs were interpreted relative to established PASS thresholds derived from previously published validation studies in primary TKA populations. For the 2011 Knee Society Score (KSS), PASS thresholds were defined as 17 (symptoms), 22 (satisfaction) and 49 (functional activities) at 1 year, and 21, 30 and 55, respectively, at 2 years [63]. For the WOMAC, a 12‐month PASS threshold of 15.8 was applied for studies reporting WOMAC on a 0–100 lower‐is‐better scale; for studies using a 0–100 higher‐is‐better convention, values were interpreted accordingly relative to domain‐specific PASS equivalents [48]. For FJS‐12, PASS thresholds of 33.3 (receiver operating characteristic [ROC]‐derived) and 77.1 (75th percentile approach) at 1 year were used [73]. For the Oxford Knee Score (OKS), PASS thresholds of 28 (6 months) and 36 (12 months) were applied [16]. PASS comparisons were performed descriptively at the study level and were limited to datasets with compatible scaling and sufficient post‐operative summary statistics. As individual patient‐level attainment data were not consistently reported, comparisons against PASS thresholds were used only to contextualize post‐operative PROM means and should not be interpreted as demonstrating the proportion of patients achieving PASS.

### Statistical analysis

Descriptive statistics were calculated and reported when available, including counts and percentages for categorical variables, means, standard deviations (SDs) and ranges for normally distributed variables, including PROMs, survivorship, complications and radiographic outcomes. The analysis of outcomes of TKA in obese patients using KA philosophy was conducted only qualitatively, as available evidence was sparse. All statistics were performed using Google Sheets (Google LLC). Weighted means were calculated where appropriate to account for differences in sample size across studies. If a study presented p‐values, the associated statistical parameters were recorded. *p* values < 0.05 were considered statistically significant.

A formal meta‐analysis was not performed due to substantial methodological and reporting heterogeneity across the included studies. PROMs were reported using multiple instruments (KSS, WOMAC, FJS‐12, OKS and SF‐12), with inconsistent scoring directions, follow‐up intervals and incomplete reporting of dispersion statistics (e.g., SDs and confidence intervals [CIs]), precluding calculation of standardized effect sizes. Survivorship outcomes were also reported using heterogeneous methods, including Kaplan–Meier estimates, cumulative revision incidence, and overall failure percentages, with variable follow‐up durations ranging from short‐term to ≥15 years, preventing reliable pooling of time‐to‐event data. Radiographic alignment outcomes were similarly heterogeneous, reported using different measurement conventions (e.g., 180° neutral HKA axis vs. deviation from 0° varus/valgus) and summary statistics (means, medians or proportions within alignment targets). Additionally, only a single study evaluated KA in obese patients, precluding comparative quantitative synthesis between alignment philosophies. Given these limitations, outcomes were synthesized descriptively and pooled only where reporting conventions were sufficiently comparable.

Survivorship outcomes were synthesized descriptively and stratified according to follow‐up duration (short‐term <5 years, mid‐term 5–10 years and long‐term ≥10 years). Given substantial heterogeneity in survivorship methodology, definitions and reporting across studies, including variation in revision endpoints (aseptic vs. all‐cause revision), analytic approaches (Kaplan–Meier estimates, cumulative revision incidence or overall failure percentages) and follow‐up duration, formal quantitative pooling of survivorship outcomes was not performed. Where studies reported failure or revision incidence rather than survivorship, survivorship was descriptively contextualized as the inverse proportion (100‐failure [%]) to facilitate qualitative comparison across studies. Survivorship values were extracted at the most recent reported time point when multiple time points were provided.

Radiographic alignment was reported using differing conventions across studies (e.g., 180° neutral mechanical axis vs. deviation from 0° varus/valgus), precluding pooled analysis.

## RESULTS

### Search results

**Figure 1:**
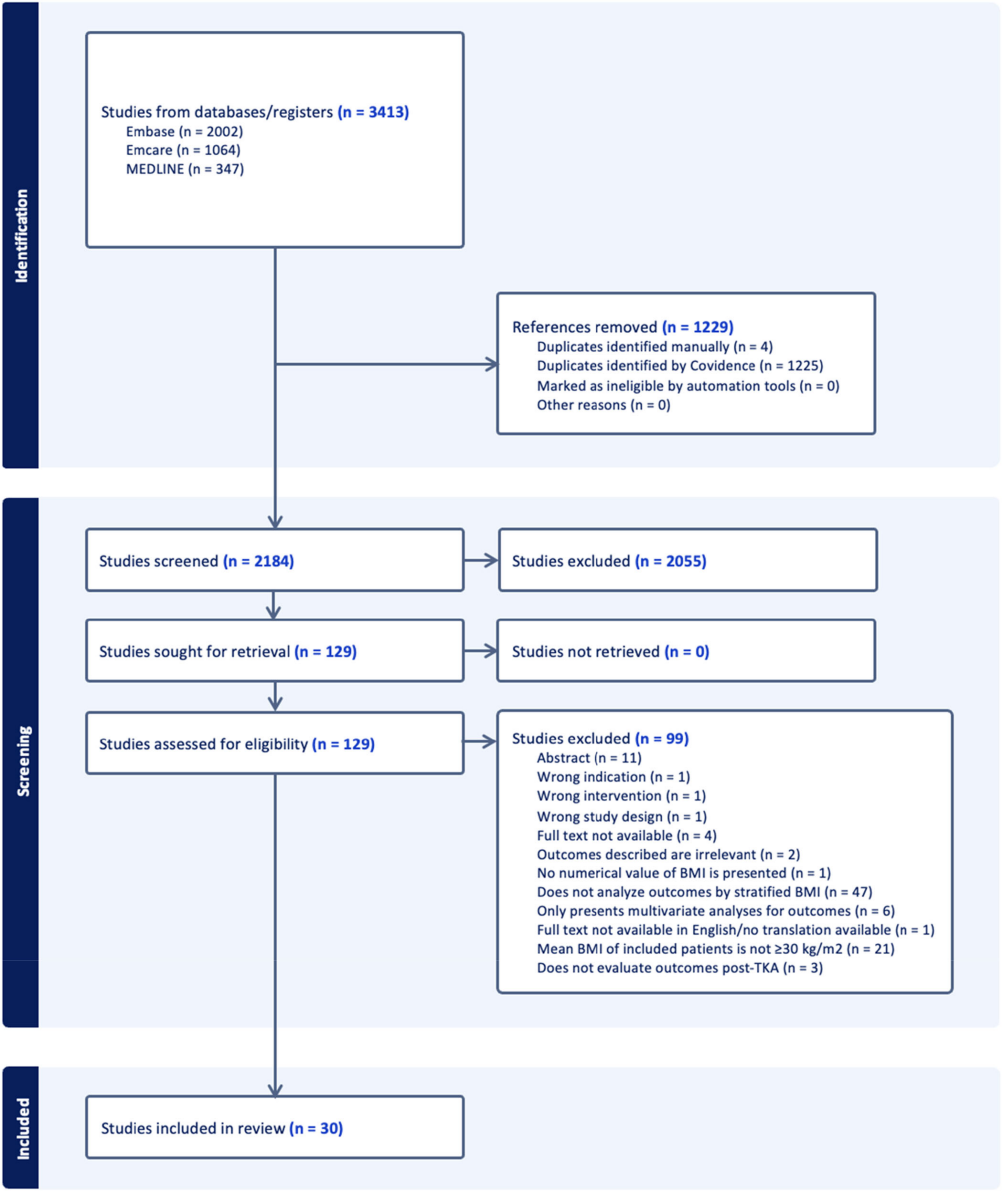
Preferred Reporting Items for Systematic Reviews and Meta‐analyses (PRISMA) flow‐chart for a systematic review investigating the outcomes of total knee arthroplasty (TKA) in obese patients (BMI ≥ 30) using mechanical alignment philosophy. BMI, body mass index.

The initial search of databases produced 3413 articles, with 1229 removed as duplicates. Thirty studies were included in the systematic review and for the qualitative synthesis of data (Figure 1) [1, 2, 3, 4, 6, 9, 10, 14, 15, 22, 24, 30, 38, 39, 42, 43, 45, 46, 47, 50, 51, 54, 56, 61, 64, 65, 68, 69, 72, 79].

### Study design and characteristics

**Figure 2:**
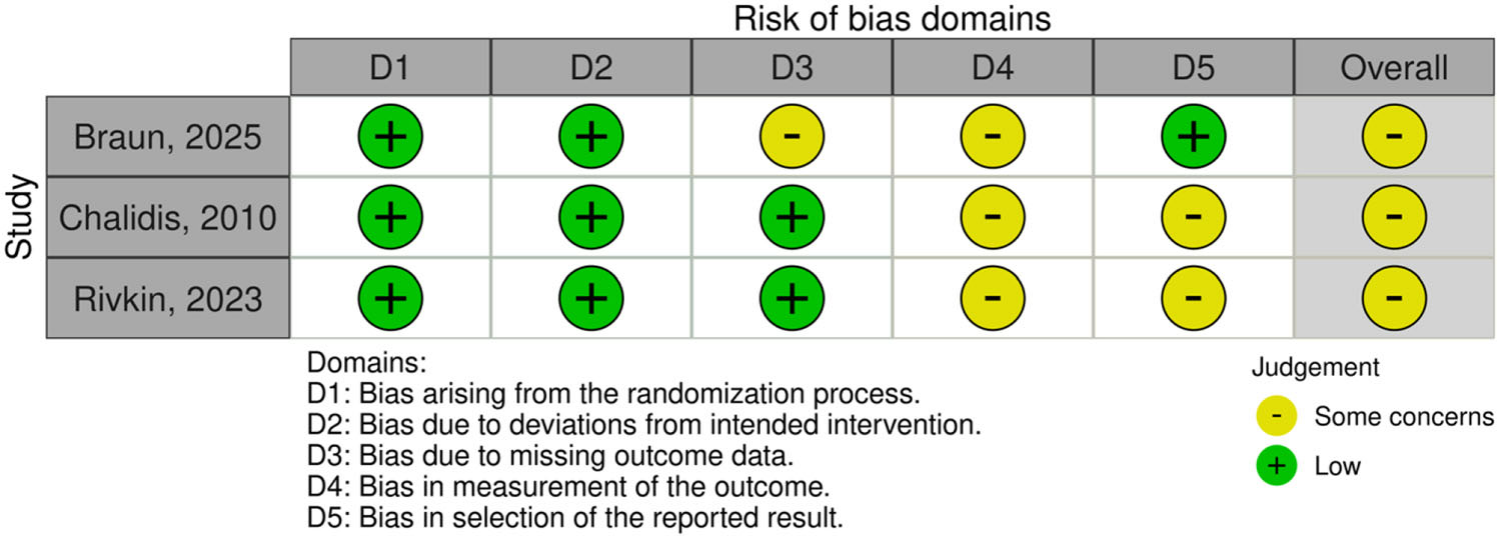
Risk of bias assessment using the ROB2 tool for randomized controlled trials. ROB2, risk of bias 2.

The articles in this review consisted of 3 Level I designs (3 RCTs), 12 Level II designs (7 prospective cohorts, 2 retrospective cohorts and 3 retrospective matched case–controls), 11 Level III designs (11 retrospective cohorts) and 3 Level IV designs (1 prospective cohort, 1 retrospective cohort and 1 retrospective case series). There was high agreement among quality assessment scores of included studies using the MINORS criteria, with an ICC = 0.8 (95% CI = 0.7–0.9). The average MINORS score for the included noncomparative studies was 11.0 (SD = 1.9), including fair quality of evidence. The average MINORS score for the included comparative studies was 18.1 (SD = 1.9), indicating good quality of evidence (Table 1). The risk of bias for the three Level I RCTs was evaluated using the ROB2 tool, and was deemed to have some concerns for risk of bias (Figure 2). Overall, the body of evidence is of moderate‐to‐high methodological quality, with consistent strengths in clearly stated aims and outcome measurement, but recurring weaknesses in bias control, blinding and study design.

**Table 1:** Study characteristics and methodological quality of included studies evaluating alignment philosophy in primary total knee arthroplasty in obese patients (BMI ≥ 30 kg/m^2^).

Author, Year	Study design	Level of evidence	Mean follow‐up, months	Number of knees	BMI classification	Mean age, years (SD) [range] {IQR}	Mean BMI	Percent female	Alignment philosophy	MINORS score
Abdel, 2015 [1]	Retrospective cohort	III	84.0	5088	<25, 25 to <30, ≥30	69.0 [31.0‐96.0]	33.0 [17.0‐69.0]	60.0%	Mechanical	17
Andrews, 2020 [2]	Retrospective cohort	III	1.5	347	30 to <35, 35 to <40, ≥40	30 to <35: 67.8 (8.3) [47.7‐87.1] 35 to <40: 63.7 (8.4) [33.7‐81.4] ≥40: 63.0 (8.4) [45.0‐80.8]	NR	30 to <35: 56.4% 35 to <40: 56.3% ≥40: 57.6%	Mechanical	9
Anwar, 2016 [3]	Prospective cohort	II	12.0	105	≥30	≥30: 65.2 [47.0‐91.0]	≥30: 35.42 [30.0‐56.0]	≥30: 54.5%	Mechanical	11
Armstrong, 2018 [4]	Prospective cohort	II	1.0	67 Obese: 43 Nonobese: 24	<30, 30 to <35, 35 to <40, ≥40	<30: 64.8 (8.3) 30 to <35: 62.6 (7.7) 35 to <40: 66.5 (10.2) ≥40: 60.2 (8.1)	<30: 27.2 (2.9) 30 to <35: 32.6 (1.7) 35 to <40: 37.5 (1.8) ≥40: 44.6 (3.7)	<30: 58.3% 30 to <35: 27.3% 35 to <40: 79.0% ≥40: 59.7%	Mechanical	10
Bansal, 2020 [6]	Retrospective cohort	IV	60.0	400	<35, ≥35	Intramedullary: 58.6 [45.0‐80.0] Extramedullary: 60.5 [45.0‐79.0]	Intramedullary: 33.8 [21.5‐44.5] Extramedullary: 32.3 [21.0‐45.0]	Intramedullary: 64.0% Extramedullary: 63.0%	Mechanical	18
Bhattacharjee, 2024 [9]	Prospective cohort	IV	12.0	42	≥30	≥30: 58.7 (8.2) [45.0‐80.0]	≥30: 33.0 [21.0‐45.0]	≥30: 63.5%	Mechanical	11
Bonnefoy‐Mazure, 2017 [10]	Prospective cohort	II	12.0	79 Obese: 34 Nonobese: 45	<25, 25 to 29.9, 30 to 34.9, ≥35, <30, ≥30	<25: 68.1 (7.8) 25‐29.9: 70.3 (6.4) 30‐34.9: 66.9 (6.8) ≥35: 67.2 (9.1) <30: 69.5 (6.9) ≥30: 67.0 (7.8)	<25: 23.7 (1.1) 25‐29.9: 27.8 (1.2) 30‐34.9: 32.1 (1.8) ≥35: 39.0 (2.4) <30: 26.4 (2.3) ≥30: 35.4 (4.0)	<25: 63.0% 25‐29.9: 58.0% 30‐34.9: 67.0% ≥35: 75.0% <30: 60.0% ≥30: 70.5%	Mechanical	19
Braun, 2025 [14]	RCT	I	60.0	158 Obese: 114 Nonobese: 44	<35, 35‐40, 40 to 45, >45	PSI: 61.0 (9.8) SOC: 62.0 (5.8)	PSI: 39.1 (6.1) SOC: 39.8 (6.5)	PSI: 70.0% SOC: 67.0%	Mechanical	NA
Chalidis, 2010 [15]	RCT	I	24.0	100	≥30	67.8 (6.9) [55.0‐82.0]	34.6 (3.8) [30.0‐46.0]	78.0%	Mechanical	NA
Elcock, 2023 [22]	Retrospective cohort	III	58.8	111	≥40	66.3 (8.8) [46.0‐82.0]	44.6 (3.6) [40.0‐58.0]	55.0%	Mechanical	17
Estes, 2013 [24]	Retrospective cohort	III	0.5	196 Obese: 84 Nonobese: 112	<35, ≥35	NR	NR	NR	Mechanical	17
Goto, 2024 [30]	Retrospective case series	IV	NR	178 Obese: 32 Nonobese: 96	>30, ≤30	>30: 74 (6.4) ≤30: 77.1 (7.4)	>30: 32.5 (2.7) ≤30: 24.9 (2.8)	NR	Mechanical	19
Ishimoto, 2024 [38]	Retrospective cohort	III	1.0	90 Obese: 21 Nonobese: 69	<25, 25 to <30, ≥30	≤25: 77.0^a^ {72.0‐79.3} 25 to <30: 75.0^a^ {73.0‐77.5} ≥30: 73.0^a^ {68.0‐77.0}	≤25: 23.4^a^ {22.1‐24.3} 25 to <30: 27.2^a^ {26.1‐28.8} ≥30: 31.6^a^ {30.8‐36.1}	≤25: 80.0% 25 to <30: 83.7% ≥30: 95.7%	Mechanical	18
Jackson, 2009 [39]	Retrospective matched case–control	II	NR	535 Obese: 153 Nonobese: 382	<30, ≥30	<30: 69.0 (5.5) [51.0‐81.0] ≥30: 69.0 (5.9) [55.0‐81.0]	<30: 25.2 (3.2) [18.0‐29.9] ≥30: 34.1 (3.8) [30.0‐46.7]	<30: 76.0% ≥30: 76.0%	Mechanical	21
Kamat, 2014 [42]	Retrospective cohort	III	60.0	287 Obese: 138 Nonobese: 149	<30, ≥30	<30 Computer navigated: 73.6 <30 standard: 72.5 ≥30 computer navigated: 71.5 ≥30 standard: 71.7	<30 Computer navigated: 23.5 <30 standard: 23.8 ≥30 computer navigated: 33.9 ≥30 standard: 34.1	<30 Computer navigated: 56.5% <30 standard: 55.0% ≥30 computer navigated: 54.7% ≥30 standard: 66.2%	Mechanical	19
Kanna, 2021 [43]	Prospective cohort	II	60.0	157 Obese: 79 Nonobese: 78	<30, ≥30	<30: 64.5 (8.8) [46.0‐82.0] ≥30: 62.2 (7.3) [51.0‐79.0]	<30: 25.6 (2.3) [18.0‐29.9] ≥30: 34 (4.2) [30.0‐44.1]	<30: 70.2% ≥30: 60.3%	Mechanical	22
Kolin, 2022 [45]	Retrospective cohort	III	24.0	292 Obese: 116 Nonobese: 116	<30, ≥30 to <35, ≥35	<30: 65.4 (8.0) ≥30 to <35: 64.7 (8.0) ≥35: 64.2 (7.0)	<30: 26.3 (2.6) ≥30 to <35: 32.1 (1.5) ≥35: 40.3 (3.8)	<30: 48.3% ≥30 to <35: 53.4% ≥35: 62.5%	Kinematically designed bicruciate‐stabilized implant with unspecified alignment strategy	16
Koutserimpas, 2025 [46]	Retrospective cohort	III	36.0	372 Obese: 134 Nonobese: 238	<30, ≥30	<30: 70.0 {64.0‐74.3} ≥30: 69.0 {63.0‐74.0}	<30: 25.8 {23.9‐27.7} ≥30: 33.6 {31.5‐35.2}	<30: 52.1% ≥30: 71.6%	Functional alignment/functional knee positioning	18
Krushell, 2007 [47]	Retrospective matched case–control	II	90.0	78 Obese: 39 Nonobese: 39	<30, >40	<30: 68.9 [39.0‐82.0] >40: 67.4 [48.0‐81.0]	<30: 25.5 [20.1‐29.2] >40: 44.2 [40.3‐53.1]	<30: 92.3% >40: 92.3%	Mechanical	16
Lai, 2022 [51]	Retrospective cohort	III	98.0	671 Obese: 156 Nonobese: 515	<25, 25 to <30, 30 to <35, 35 to <40, ≥40	<25: 67.4 (8.1) 25 to <30: 66.4 (6.7) 30 to <35: 64.1 (5.9) 35 to <40: 60 (3.6) ≥40: 64 (4.2)	<25: 22.4 (1.9) 25 to <30: 27.2 (1.3) 30 to <35: 31.6 (1.4) 35 to <40: 35.7 (0.4) ≥40: 44.1 (1.0)	<25: 78.1% 25 to <30: 81.3% 30 to <35: 84.3% 35 to <40: 86.5% ≥40: 75.0%	Mechanical	17
Lai, 2024 [50]	Retrospective cohort	III	6.0	156	≥30	Neutral: 72.8 (6.7) Mild varus: 74.4 (6.7)	Neutral: 31.5 (1.4) Mild varus: 31.5 (1.4)	Neutral: 84.9% Mild varus: 89.1%	Mechanical	17
Li, 2018 [54]	Prospective cohort	II	NR	406 Obese: 183 Nonobese: 223	<25, 25 to 30, >30	67.5 (10.5) [33.0‐93.0]	30.0 (5.3) [19.5‐48.7]	65.8%	Mechanical	14
Lizaur‐Utrilla, 2014 [56]	Prospective cohort	II	84.0	342 Obese: 171 Nonobese: 171	<25, 25 to <30, 30 to <35, 35 to <40, ≥40	<30: 70.7 [45.0‐83.0] ≥30: 70.2 [43.0‐81.0]	<30: 26.0 [17.5‐29.6] ≥30: 36.0 [31.1‐53.1]	<30: 76.0% ≥30: 76.0%	Mechanical	21
Mont, 1996 [61]	Retrospective matched case‐control	II	Nonobese: 62.0 Obese: 65.0	100 Nonobese: 50 Obese: 50	<30, ≥40	<30: 58.0 [30.0‐76.0] ≥40: 61.0 [30.0‐74.0]	NR	<30: 18.0% ≥40: 18.0%	Mechanical	20
Ojard, 2018 [64]	Retrospective cohort	III	NR	251 Obese: 149 Nonobese: 102	<30, ≥30	<30: 71.4 [52.0‐88.0] ≥30: 63.9 [40.0‐85.0]	<30: 26.5 [20.2‐29.9] ≥30: 37.0 [30.1‐53.0]	<30: 58.0% ≥30: 64.0%	Mechanical	16
Puah, 2020 [65]	Prospective cohort	II	87.7	101 Obese: 23 Nonobese: 78	>23, >27.5, >30	65.3 (6.9)	27.2 (4.1) [18.6‐40.0]	83.2%	Mechanical	21
Ritter, 2011 [68]	Retrospective cohort	II	91.3	6070	≥30	70.1 (8.6) [21.0‐93.0]	30.2 (5.6) [16.5‐64.3]	61.0%	Mechanical	16
Rivkin, 2023 [69]	RCT	I	NR	60 Conventional: 30 Computer‐ assisted: 30	>30	Conventional: 70.1 Computer‐assisted: 66.9	Conventional: 34.4 Computer‐assisted: 35.6	Conventional: 63.3% Computer‐assisted: 70.0%	Mechanical	NA
Shetty, 2014 [72]	Retrospective cohort	II	48.0	1155 Obese: 520 Nonobese: 635	<30, ≥30, >40	<30: 67.3 (7.8) [42.0‐86.0] ≥30: 65.5 (7.7) [46.0‐94.0] >40: 65.4 (7.5) [50.0‐78.0]	<30: 26.4 (2.5) [15.2‐29.9] ≥30: 34.9 (4.4) [30.0‐54.1] >40: 44.7 (4.0) [40.3‐54.1]	NR	Mechanical	17
Yoo, 2018 [79]	Retrospective cohort	III	83.1	371 Obese: 78 Nonobese: 293	<25, 25 to <30, ≥30	<25: 69.2 (4.8) [59.0‐80.0] 25 to <30: 69.7 (5.3) [55.0‐80.0] ≥30: 68.7 (5.7) [53.0–79.0]	<25: 23.1 (1.4) 25 to <30: 27.2 (1.5) ≥30: 32.5 (2.1)	<25: 91.2% 25 to <30: 92.2% ≥30: 97.4%	Mechanical	17

Abbreviations: IQR, interquartile range; NA, not applicable; NR, not reported; PSI, patient‐specific instrumentation; RCT, randomized controlled trial; SD, standard deviation; SOC, standard of care instrumentation.

^a^ Median values.

### Systematic review of outcomes of TKA in obese patients by alignment philosophy

A total of 14,662 knees in obese patients (BMI ≥ 30 kg/m^2^), with a mean age of 69.3 (SD = 8.6) years (*n* = 7687) and a mean post‐operative follow‐up of 91.1 (SD = 45.3) months (*n* = 6171) underwent TKA with MA and were included in the quantitative synthesis. One study evaluated FA/functional knee positioning, including 134 obese knees (BMI ≥ 30 kg/m^2^) with a median age of 69.0 (interquartile range [IQR] = 63.0–74.0) and median follow‐up of 30.0 (IQR = 26.0–36.0) months. Another study investigated the outcomes of TKA in obese patients with Journey II bicruciate‐stabilized, kinematically designed implant with unspecified alignment, which included 88 obese patients (BMI ≥ 30 to <35 kg/m^2^) with a mean age of 64.7 (SD = 8.0), 88 severely obese patients (BMI ≥ 35 kg/m^2^) with a mean age of 64.2 (SD = 7.0) years, and 116 patients with <30 kg/m^2^ with a mean age of 65.4 (SD = 8.0) years, all with a minimum follow‐up of 24 months. Importantly, the available evidence is highly imbalanced with respect to alignment philosophy: of the included 14,796 obese knees, only 204 knees underwent a kinematic implant with unknown alignment; consequently, the available literature does not permit meaningful comparison between alignment strategies in obese populations, and findings related to non‐MA approaches should be interpreted cautiously. This imbalance substantially limits the ability of the present review to determine whether alignment philosophy independently influences outcomes in obese TKA.

#### PROMs

Across the abstracted studies using MA alignment philosophy, reporting of PROMs was heterogeneous with respect to instruments (e.g., KSS, WOMAC, OKS, Short‐Form Questionnaire [SF‐12], FJS‐12, Hospital for Special Surgery Knee‐Rating Scale [HSS]‐derived scores), time points (early recovery to mid‐/long‐term) and BMI stratification methods (binary BMI < 30 vs. ≥30 kg/m^2^, BMI < 35 vs. ≥35 kg/m^2^, multi‐categorical strata or obese‐only cohorts). Where reported, most studies demonstrated substantial post‐operative improvement in knee‐specific outcomes within obese cohorts (e.g., improved KSS and WOMAC domains), although some studies identified small but statistically significant decrements in longer‐term function or symptom scores in obese versus non‐obese comparators (e.g., lower WOMAC and KSS function at 60 months in BMI ≥ 30 vs. BMI < 30 kg/m^2^) (Table 2) [9, 10, 14, 39, 46, 47, 50, 51, 56, 65, 79].

**Table 2:** Post‐operative patient‐reported outcome measures (PROMs) following primary total knee arthroplasty in obese patients, stratified by alignment philosophy and BMI category.

Author, Year	Number of knees	Knee Society Score (KSS) [range] {IQR} *Higher scores = better outcome	WOMAC [range] {IQR}	Forgotten Joint Score 12 (FJS‐12) [range] {IQR} *Higher scores = better outcome	Oxford Knee Score (OKS) [range] {IQR} *Higher scores = better outcome	Hospital for Special Surgery (HSS) Knee Score [range] {IQR} *Higher scores = better outcome	Short‐Form Questionnaire (SF‐12) [range] {IQR} *Higher scores = better outcome
Bhattacharjee, 2024 [9]	42	KSS Total, mean (3 mo.) ≥30: 70.7 [52.0–84.0] KSS Total, mean (6 mo.) ≥30: 80.9 [68.0–92.0]	NR	NR	NR	NR	NR
Bonnefoy‐Mazure, 2017 [10]	79 Obese: 34 Nonobese: 45	NR	*Higher scores = better outcome WOMAC Pain, mean (12 mo.) <30: 82.7 ± 15.5 ≥30: 80.7 ± 15.5 WOMAC Function, mean (12 mo.) <30: 80.9 ± 15.7 ≥30: 76.4 ± 18.7	NR	NR	NR	SF‐12 Physical Component, mean (12 mo.) <30: 45.2 ± 7.6 ≥30: 41.2 ± 9.8 SF‐12 Mental Component, mean (12 mo.) <30: 49.3 ± 10.0 ≥30: 46.1 ± 12.3
Braun, 2025 [14]	158 Obese: 114 Nonobese: 44	KSS Total, mean (60 mo.) <35: 86.9 ± 11.2 ≥35: 85.4 ± 12.6 *p* = 0.48 KSS Function, mean (60 mo.) <35: 82.1 ± 16.8 ≥35: 79.3 ± 18.4 *p* = 0.41	*Higher scores = better outcome WOMAC, mean (60 mo.) <35: 21.6 ± 14.3 ≥35: 23.1 ± 15.1 *p* = 0.62	NR	NR	NR	SF‐12 Physical Component, mean (60 mo.) <35: 44.8 ± 9.7 ≥35: 42.9 ± 10.1 *p* = 0.29 SF‐12 Mental Component, mean (60 mo.) <35: 52.3 ± 8.4 ≥35: 51.7 ± 9.1 *p* = 0.67
Chalidis, 2010 [15]	100	KSS Pain, median (24 mo.) ≥30: 90.0 [70.0–100.0] KSS Function, median (24 mo.) ≥30: 85.0 [65.0–100.0]	NR	NR	NR	NR	NR
Elcock, 2023 [22]	111	NR	NR	NR	OKS, mean (58.8 mo.) SK ≥ 40: 11.9 ± 10.1 UBP ≥ 40: 12.9 ± 10.8 *p* = 0.52	NR	NR
Jackson, 2009 [39]	535 Obese: 153 Nonobese: 382	NR	NR	NR	NR	HSS, mean (110.5 mo.) ≥ 30: 83.8 ± 9.3 >30: 87.4 ± 8.6 *p* < 0.05 HSS Pain, mean (110.5 mo.) ≥30: 26.8 ± 6.8 >30: 27.3 ± 5.1 *p* = 0.68 HSS Function, mean (110.5 mo.) ≥30: 15.0 ± 3.2 >30: 16.7 ± 3.5 *p* < 0.01 HSS Range of Motion, mean (110.5 mo.) ≥30: 13.4 ± 2.1 >30: 14.6 ± 2.0 *p* < 0.01	NR
Kolin, 2022 [45]	292 Obese: 116 Nonobese: 116	KSS Total, mean (24 mo.) <30: 84.4 ± 3.2 ≥30 to <35: 83.7 ± 3.3 ≥35: 84.2 ± 5.0	NR	NR	NR	NR	NR
Koutserimpas, 2025 [46]	372 Obese: 134 Nonobese: 238	KSS Knee, mean (24 mo.) ≥30: 95.0 {90.0–100.0} <30: 94.5 {90.0–100.0} *p* = 0.62 KSS Function, mean (24 mo.) ≥30: 90.0 {90.0–100.0} <30: 90.0 {90.0–100.0} *p* = 0.47	NR	FJS‐12, mean (24 mo.) ≥30: 86.0 {75.0–94.0} <30: 84.0 {65.0–92.0} *p* = 0.16	NR	NR	NR
Krushell, 2007 [47]	78 Obese: 39 Nonobese: 39	KSS Function, mean (90 mo.) >40: 44.0 <30: 64.0 *p* < 0.005 KSS Total, mean (90 mo.) >40: 91.0 <30: 94.0	NR	NR	NR	NR	NR
Lai, 2022 [51]	671 Obese: 156 Nonobese: 515	KSS Total, mean (98 mo.) <25: 114.3 ± 35.3 25–29.9: 90.7 ± 19.4 30–34.99: 84.5 ± 10.3 35–39.9: 81.6 ± 8.8 >40: 69.4 ± 24.3 *p* = 0.001 KSS Function, mean (98 mo.) <25: 86.4 ± 8.7 25–29.9: 73.9 ± 16.1 30–34.99: 78.9 ± 22.0 35–39.9: 72.6 ± 12.2 >40: 66.2 ± 16.8 *p* = 0.58	*Lower scores = better outcome WOMAC, mean (98 mo.) <25: 11.8 ± 13.1, 25–29.9: 12.0 ± 8.6 30–34.99: 15.8 ± 10.4 35–39.9: 16.2 ± 15.8 >40: 28.5 ± 14.8 *p* = 0.022	FJS‐12, mean (98 mo.) <25: 81.3 ± 15.1 25–29.9: 77.5 ± 21.9 30–34.99: 72.5 ± 27.3 35–39.9: 62.4 ± 34.7 >40: 54.2 ± 31.3 *p* = 0.032	NR	NR	NR
Lai, 2024 [50]	156	KSS Total, mean (6 mo.) Neutral alignment: 83.7 ± 10.6 Mild varus: 87.3 ± 8.1 *p* = 0.16 KSS Function, mean (6 mo.) Neutral alignment: 75.7 ± 20.1 Mild varus: 78.3 ± 17.4 *p* > 0.99	*Lower scores = better outcome WOMAC, mean (6 mo.) Neutral alignment: 15.0 ± 14.2 Mild varus: 8.3 ± 8.6 *p* = 0.009	FJS‐12, mean (6 mo.) Neutral alignment: 70.2 ± 30.0 Mild varus: 86.0 ± 15.6 *p* = 0.002	NR	NR	NR
Lizaur‐Utrilla, 2014 [56]	342 Obese: 171 Nonobese: 171	KSS Total, mean (60 mo.) ≥30: 86.8 <30: 88.5 *p* = 0.11 KSS Function, mean (60 mo.) ≥30: 88.6 <30: 91.7 *p* = 0.013	*Higher scores = better outcome WOMAC Pain, mean (60 mo.) ≥30: 90.7 <30: 92.4 *p* = 0.16 WOMAC, mean (60 mo.) ≥30: 92.5 <30: 94.5 *p* = 0.019	NR	NR	NR	SF‐12 Physical Component, mean (60 mo.) ≥30: 88.5 <30: 90.1 *p* = 0.13 SF‐12 Mental Component, mean (60 mo.) ≥30: 92.1 <30: 94.3 *p* = 0.28
Puah, 2020 [65]	101 Obese: 23 Nonobese: 78	NR	NR	NR	OKS, mean (24 mo.) <30: 18.3 ± 4.7 ≥30: 20.7 ± 7.8 *p* = 0.70	NR	NR
Yoo, 2018 [79]	371 Obese: 78 Nonobese: 293	KSS Total, mean (3 mo.) <25: 91.8 ± 6.0 25–30: 92.0 ± 7.3 ≥30: 93.3 ± 4.3 *p* = 0.31 KSS Total, mean (12 mo.) <25: 95.4 ± 4.7 25–30: 94.1 ± 6.1 ≥30: 93.8 ± 5.0 *p* = 0.14 KSS Total, mean (60 mo.) <25: 91.2 ± 7.2 25–30: 91.5 ± 7.4 ≥30: 90.7 ± 7.4 *p* = 0.79	NR	NR	NR	NR	NR

Abbreviations: HSS, Hospital for Special Surgery Knee Score; IQR, interquartile range; mo., months; NR, not reported; SF‐12, Short Form 12 Health Survey; SK, standard keeled tibial baseplate; UBP, universal tibial baseplate; WOMAC, Western Ontario and McMaster Universities Osteoarthritis Index.

Kolin et al., who investigated the impact of kinematic implant with unknown alignment philosophy, reported significant pre‐ to post‐operative improvements in KSS and KOOS JR scores at ≥2‐year follow‐up in both obese (BMI ≥ 30 to <35 kg/m^2^) and severely obese (BMI ≥ 35 kg/m^2^) patients, with low post‐operative VAS pain scores in both groups. Post‐operative KSS (83.7) and KOOS JR (88.5) were comparable between BMI strata. No statistically significant differences were observed between BMI groups at baseline, follow‐up or in the magnitude of improvement (Table 2).

Koutserimpas et al., who investigated the impact of FA philosophy using image‐based robotic TKA, reported comparable post‐operative PROMs between obese and non‐obese patients at 2‐year follow‐up [46]. Post‐operative KSS‐knee scores were similar between BMI strata (BMI ≥ 30 kg/m^2^ 95.0 [90.0–100.0] vs. BMI < 30 kg/m^2^ 94.5 [90.0–100.0]; *p* = 0.62), as were KSS‐function scores (BMI ≥ 30 kg/m^2^ 90.0 [90.0–100.0] vs. BMI < 30 kg/m^2^ 90.0 [90.0–100.0]; *p* = 0.47). FJS‐12 scores were also comparable between groups (*p* = 0.16), suggesting similar patient‐reported functional outcomes between obese and non‐obese cohorts following FA‐TKA.

#### PROMs—Contextualization relative to PASS attainment

Post‐operative PROMs in obese cohorts were descriptively contextualized against established PASS thresholds when scale direction and reporting format permitted comparison. However, as included studies generally reported group‐level post‐operative means rather than patient‐level PASS attainment, these comparisons should be interpreted as contextual rather than demonstrative of the proportion of patients achieving an acceptable symptom state.

Across the 10 studies reporting PROM differences by BMI [10, 14, 39, 45, 46, 47, 51, 56, 65, 79], obese cohorts frequently achieved PROM values consistent with an acceptable symptom state. Reported post‐operative KSS total scores at mid‐ to long‐term follow‐up (means 85.4–90.7 at 60 months in obese strata) [14, 56, 79] and KSS Function scores (means 66.2–84.9 in obese strata) were well above published PASS thresholds for symptoms (17–21), satisfaction (22–30) and functional activities (49–55), indicating that most obese cohorts would be expected to meet an acceptable symptom state.

Using a 0–100 higher‐is‐better WOMAC scale (PASS threshold = 84.2 on higher‐is‐better or 15.8 on lower‐is‐better equivalent pain/function domains), Lizaur‐Utrilla et al. reported obese WOMAC totals of 92.5 and pain scores of 90.7 at 60 months [56], consistent with PASS attainment. Lai et al. demonstrated that obese patients with BMI 30–39.9 kg/m^2^ had mean WOMAC totals of 15.8–16.2 on a 0–100 lower‐is‐better scale at 98 months, approximating or slightly exceeding the PASS threshold of 15.8, while BMI > 40 kg/m^2^ fell short at a mean WOMAC score of 28.5 [51].

For the FJS‐12, obese means of 54.2–86.0 at 24–98 months [46, 50, 51] frequently exceeded the ROC‐derived PASS threshold of 33.3 and, in several cohorts (e.g., FJS‐12 = 86.0 at 24 months), also surpassed the more stringent 75th percentile threshold of 77.1. Reported OKS means for obese cohorts at 24–59 months (20.7 [65] and 11.9–12.9 [22], depending on scoring direction) were below contemporary PASS cutoffs of approximately 28–36 at 6–12 months, suggesting inconsistent attainment depending on instrument scaling. Given the absence of patient‐level responder analyses, these findings should be interpreted only as providing clinical context for post‐operative PROM performance rather than evidence of true PASS attainment rates.

#### PROMs—Frequency of statistically significant obese versus non‐obese differences across alignment philosophies

Across the 30 included studies, 14 directly compared PROMs between obese and non‐obese patients following TKA [4, 10, 38, 39, 42, 43, 45, 46, 47, 51, 56, 61, 65, 79]. Of these, 9/14 studies (64.3%) did not report statistically significant differences in PROMs between obese and non‐obese cohorts at final follow‐up, suggesting broadly comparable patient‐reported functional and pain outcomes across BMI categories [4, 38, 42, 43, 45, 46, 61, 65, 79]. Seven of these studies investigated MA alignment on post‐operative outcomes [4, 38, 42, 43, 61, 65, 79], while one study [45] investigated a kinematic implant with unspecified alignment on outcomes, and one study [46] investigated FA alignment on outcomes. Ishimoto et al. observed no significant group effect for WOMAC pain (obese vs. normal weight *p* = 0.280) or WOMAC function (*p* = 0.792), and no significant BMI‐group‐by‐time interaction was identified (all *p* > 0.400), indicating comparable short‐term recovery trajectories across BMI groups [38]. Likewise, Koutserimpas et al. reported similar post‐operative PROMs between BMI ≥ 30 and <30 cohorts, including KSS‐knee (*p* = 0.62), KSS‐function (*p* = 0.47) and FJS (*p* = 0.16) [46]. Similarly, the one paper investigating kinematic implant with unknown alignment by Kolin et al. observed no differences in PROMs at baseline or follow‐up (all *p* values > 0.10), and no difference in improvement was noted for KSS (*p* = 0.21) or KOOS‐JR (*p* = 0.62) [45].

In contrast, 5/14 studies (35.7%) reported at least one statistically significant difference between obese and non‐obese patients [10, 39, 47, 51, 56]. Where differences were observed, they most commonly reflected slightly lower post‐operative functional scores or smaller improvements in obese patients; however, these findings are not consistent across outcome instruments or time points. Obese patients in the study by Lizaur‐Utrilla et al. demonstrated lower KSS function (*p* = 0.013) and WOMAC function scores (*p* = 0.019) at final follow‐up [56]. Similarly, in their 2022 paper, Lai et al. reported WOMAC scores that differed significantly across BMI groups (*p* = 0.022), with higher BMI associated with worse PROM performance [51]. Additionally, Bonnefoy‐Mazure et al. demonstrated baseline differences in WOMAC pain and function (both *p* < 0.001), and a significant difference in WOMAC pain gain (*p* = 0.011), although change in WOMAC function narrowly missed significance (*p* = 0.055) [10].

#### Survivorship

Survivorship and revision outcomes were reported heterogeneously as revision‐free survival or cumulative revision incidence; values are reported as described in the original studies, which include Kaplan‐Meier revision‐free survival, cumulative revision incidence, and overall percentages. Survivorship outcomes in obese patients undergoing mechanically aligned TKA were reported in five studies [1, 14, 39, 56, 68], with follow‐up durations ranging from short‐term (5 years) to long‐term (15 years). Due to heterogeneity in survivorship definitions, time horizons and reporting formats, survivorship outcomes were not pooled, and survivorship outcomes were synthesized descriptively (Table 3). Where studies reported revision or failure incidence rather than survivorship, values were contextualized descriptively to facilitate comparison across studies.

**Table 3:** Revision‐free survivorship and revision incidence following mechanically aligned primary total knee arthroplasty in obese patients across short‐, mid‐ and long‐term follow‐up, reported according to original study definitions.

Author, Year	Number of knees	BMI classification	Outcome	Methodology	Short‐term (<5 years) [range] {95% CI}	Mid‐term (5–10 years) [range] {95% CI}	Long‐term (≥10 years) [range] {95% CI}
Abdel, 2015 [1]	5088	<25, 25 to <30, ≥30	Aseptic revision incidence	Kaplan–Meier	<35: 0.5% {0.3%–0.6%} ≥35: 1.3% {0.9%–1.6%}	NR	<35: 2.2% {1.7%–2.7%} ≥35: 4.3% {3.3%–5.3%}
Braun, 2025 [14]	158 Obese: 114 Nonobese: 44	<35, ≥35	All‐cause revision rate	Per cent revision incidence	NR	PSI: 6.3% SOC: 2.6%	NR
Jackson, 2009 [39]	535 Obese: 153 Nonobese: 382	<30, ≥30	All‐cause revision‐free	Kaplan–Meier	NR	NR	<30: 96.4% {92.0%–99.0%} ≥30: 98.0% {95.9%–99.0%}
Lizaur‐Utrilla, 2014 [56]	342 Obese: 171 Nonobese: 171	<25, 25 to <30, 30 to <35, 35 to <40, ≥40	All‐cause revision‐free	Kaplan–Meier	NR	<30: 91.8% {84.3%–100.0%} ≥30: 88.9% {79.4%–98.5%}	NR
Ritter, 2011 [68]	6070	>30	Overall failure incidence	Overall per cent failure	NR	0.9%	NR

Abbreviations: 95% CI, 95% confidence interval; BMI, body mass index; NR, not reported; PSI, patient‐specific instrumentation; SOC, standard of care.

Short‐term (<5 years) cumulative aseptic revision incidence in obese patients ranged from 0.5% (95% CI = 0.3–0.6) to 1.3% (95% CI = 0.9–1.6), depending on BMI category [1], corresponding to 98.7% to 99.5% freedom from aseptic revision when interpreted as the inverse proportion. Mid‐term (5–10 years) survivorship was reported in two studies, with Jackson et al. reporting a mid‐term revision‐free survivorship of 88.9% in patients with BMI ≥ 30 kg/m^2^ [39]. Braun et al. reported a mid‐term all‐cause per cent revision incidence of 6.3% in obese populations who received PSI in TKA and 2.6% in obese populations who received standard of care in TKA [14]. Long‐term (≥10 years) aseptic revision‐free survivorship was reported in two studies, with Jackson et al. reporting an all‐cause revision‐free survivorship of 98.0% in patients with BMI ≥ 30 kg/m^2^ [39]. Abdel et al. reported long‐term cumulative aseptic revision incidence in obese patients ranged from 2.2% (95% CI = 1.7–2.7) to 4.3% (95% CI = 3.3–5.3) [1]. One study reported survivorship as an overall percentage of failure without specifying a time point, documenting 0.9% TKA failure in patients with BMI > 30 kg/m^2^ [68].

#### Reasons for revision

**Figure 3:**
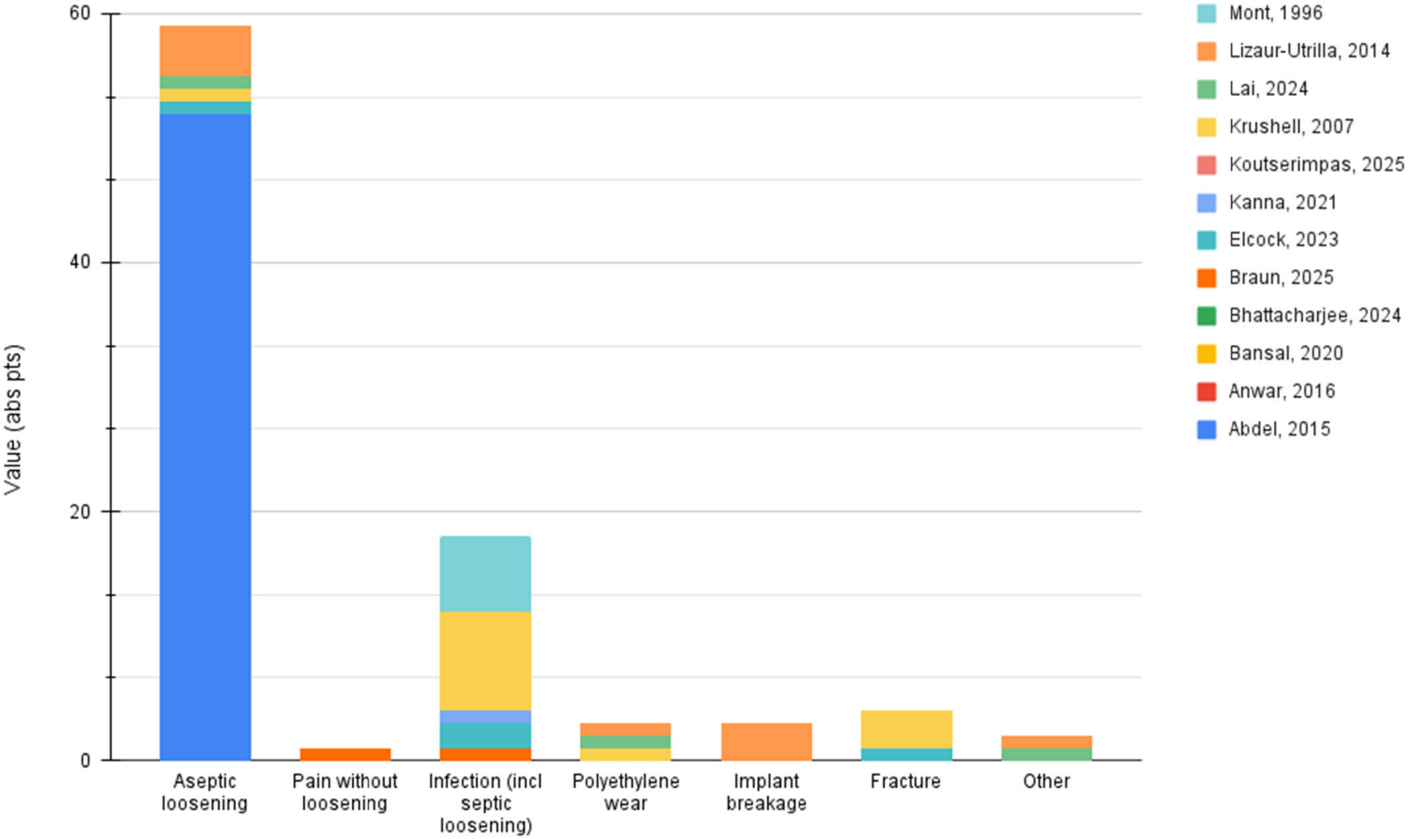
Distribution of reported causes of revision following total knee arthroplasty in obese patient cohorts across included studies. These data are descriptive and should not be interpreted as obesity‐specific revision patterns, as direct comparison with non‐obese revision distributions was inconsistently reported. Abs pts, absolute points; incl, including.

Reporting of reasons for revision was inconsistent across studies, with some providing cumulative revision rates and revision indications stratified by BMI, and others reporting overall revision rates without indication breakdown (Table 4). Across 13,490 obese knees, total indications for revision included aseptic loosening (*n* = 62), infection or septic loosening (*n* = 22), periprosthetic fracture (*n* = 4), pain without loosening (*n* = 2), polyethylene wear (*n* = 2), implant breakage (*n* = 5) and other (*n* = 3) [1, 3, 6, 9, 14, 22, 43, 46, 47, 50, 56, 61] (Figure 3). Cumulative revision rates in obese strata range from 0.0% in short follow‐up obese‐only cohorts [3, 6, 9, 46] to 3%–5% in studies reporting BMI‐stratified revision rates at mid‐term follow‐up [14, 22, 56].

**Table 4:** Indications for revision following primary total knee arthroplasty in obese patients (BMI ≥ 30 kg/m^2^), stratified by study and BMI category.

Author, Year	Number of knees	Aseptic loosening, *n*	Pain without loosening, *n*	Infection (includes septic loosening), *n*	Polyethylene wear, *n*	Implant breakage, *n*	Fracture, *n*	Other, *n*	Total, *n*
Abdel, 2015 [1]	5088	52	0	0	0	0	0	0	52
Anwar, 2016 [3]	105	0	0	0	0	0	0	0	0
Bansal, 2020 [6]	400	0	0	0	0	0	0	0	0
Bhattacharjee, 2024 [9]	42	0	0	0	0	0	0	0	0
Braun, 2025 [14]	158 Obese: 114 Nonobese: 44	<35: 0 ≥35: 0	<35: 1 ≥35: 1	<35: 1 ≥35: 1	<35: 0 ≥35: 0	<35: 0 ≥35: 0	<35: 0 ≥35: 0	<35: 0 ≥35: 0	<35: 2 ≥35: 2
Elcock, 2023 [22]	111	1	0	2	0	0	1	0	4
Kanna, 2021 [43]	157 Obese: 79 Nonobese: 78	0	0	1	0	0	0	0	1
Koutserimpas, 2025 [46]	372 Obese: 134 Nonobese: 238	<30: 0 ≥30: 0	<30: 0 ≥30: 0	<30: 0 ≥30: 0	<30: 0 ≥30: 0	<30: 0 ≥30: 0	<30: 0 ≥30: 0	<30: 0 ≥30: 0	<30: 0 ≥30: 0
Krushell, 2007 [47]	78 Obese: 39 Nonobese: 39	1	0	8	1	0	3	0	13
Lai, 2024 [50]	156	1	0	0	1	0	0	1	3
Lizaur‐Utrilla, 2014 [56]	342 Obese: 171 Nonobese: 171	<30: 1 ≥30: 4	<30: 0 ≥30: 0	<30: 1 ≥30: 0	<30: 0 ≥30: 1	<30: 2 ≥30: 3	<30: 0 ≥30: 0	<30: 1 ≥30: 1	<30: 5 ≥30: 9
Mont, 1996 [61]	100 Nonobese: 50 Obese: 50	<30: 2 ≥40: 0	<30: 0 ≥40: 0	<30: 1 ≥40: 6	<30: 0 ≥40: 0	<30: 0 ≥40: 0	<30: 0 ≥40: 0	<30: 0 ≥40: 0	<30: 3 ≥40: 6
Overall, *n*	7001	62	2	22	2	5	4	3	100

Although aseptic loosening and infection were the most frequently reported revision indications in obese cohorts, interpretation of these findings should be made cautiously, as relatively few studies directly compared revision patterns between obese and non‐obese patients. Further, the distribution of revision indications observed in obese patients broadly resembles commonly reported causes of failure in general primary TKA populations, where aseptic loosening and periprosthetic joint infection remain among the leading reasons for revision. Consequently, the present review cannot determine whether the observed revision distribution is specific to obesity or reflects expected revision patterns following TKA more generally.

#### Radiographic outcomes

Radiographic outcomes included global limb alignment measures (most commonly HKA angle, variably defined and scaled), femorotibial angle measures, and component alignment (coronal and sagittal femoral/tibial alignment), with some studies additionally reporting the proportion of cases within predefined alignment targets. These radiographic outcomes were reported in several studies but not pooled due to heterogeneity in measurement convention, summary statistics and reporting formats (Table 5) [47, 50, 51, 56, 79].

**Table 5:** Post‐operative radiographic alignment and component positioning outcomes following primary total knee arthroplasty in obese patients, reported by alignment convention and BMI strata.

Author, Year	Number of knees	Hip–knee–ankle (HKA) angle, degrees [range] {IQR}	Tibial component alignment (TCA), degrees [range] {IQR}	Femorotibial alignment (FTA), degrees [range] {IQR}	Mechanical medial proximal tibial angle (mMPTA), degrees [range] {IQR}
Abdel, 2015 [1]	5088	NR	NR	FTA, mean (84 mo.) No revision: 5.3 ± 0.3 Revision: 6.8 ± 0.4	NR
Andrews, 2020 [2]	347	NR	TCA, mean (1.5 mo.) Males: 0.3 ± 1.4 [−4.0 to 4.0] Females: 0.2 ± 1.4 [−5.0 to 5.0]	NR	NR
Anwar, 2016 [3]	105	HKA, mean (12 mo.) ≥30: 2.6 ± 2.1	TCA, mean (12 mo.) ≥30 89.2 ± 1.4°	NR	NR
Braun, 2025 [14]	158 Obese: 114 Nonobese: 44	NR	NR	FTA, mean (60.0 mo.) <35: 179.1 ± 2.6 ≥35: 178.7 ± 2.9 *p* = 0.41	mMPTA, mean (60.0 mo.) <35: 89.1 ± 2.3 ≥35: 88.8 ± 2.5 *p* = 0.47
Goto, 2024 [30]	178 Obese: 32 Nonobese: 96	HKA, mean (follow‐up NR) ≤30: 0.6 ± 2.8 >30: −0.3 ± 2.3 *p* = 0.069	NR	NR	mMPTA, mean (follow‐up NR) ≤30: 89.9 ± 2.4 >30: 90.6 ± 1.9 *p* = 0.76
Kanna, 2021 [43]	157 Obese: 79 Nonobese: 78	HKA, 180° neutral convention, mean (60 mo.) <30: 179.3 ± 1.9 [175.0–185.0] ≥30: 179.0 ± 1.5 [175.0–184.0] *p* = 0.250	TCA, mean (60.0 mo.) <30: 90.6 ± 1.4 [86.0–94.0] ≥30: 90.2 ± 1.7 [86.0–94.0] *p* = 0.13	NR	NR
Koutserimpas, 2025 [46]	372 Obese: 134 Nonobese: 238	HKA, 180° neutral convention, mean (24 mo.) ≥30: 178.0 {176.0–180.0} <30: 179.0 {177.0–180.8} *p* = 0.13	NR	NR	mMPTA, mean (24 mo.) ≥30: 88.0 {86.0–89.0} <30: 89.0 {87.0–90.0} *p* = 0.05
Krushell, 2007 [47]	78 Obese: 39 Nonobese: 39	NR	NR	FTA, mean (90 mo.) <30: 5.5 >40: 2.6	NR
Lai, 2022 [51]	671 Obese: 156 Nonobese: 515	HKA, mean (98 mo.) <25: −2.7 ± 2.3 25–29.9: −2.8 ± 2.2 30–34.99: −4.4 ± 2.3 35–39.9: −5.9 ± 3.4 >40: −5.9 ± 0.2	NR	NR	NR
Lai, 2024 [50]	156	HKA, mean (6 mo.) Neutral: −1.0 ± 0.1 Mild varus: −4.3 ± 0.1 Severe varus: −7.3 Valgus: 3.8 ± 0.3	NR	NR	NR
Lizaur‐Utrilla, 2014 [56]	342 Obese: 171 Nonobese: 171	NR	NR	FTA, mean (84 mo.) ≥30: 5.6 [0.0–12.0] <30: 5.8 [0.0–12.0] *p* = 0.38	NR
Ritter, 2011 [68]	6070	NR	TCA, mean (91.3 mo.) >30: 90.4 ± 2.4 [78.0–102.0]	NR	NR
Rivkin, 2023 [69]	60 Conventional: 30 Computed‐assisted: 30	HKA, mean (follow‐up NR) Computed‐assisted: 2.8 Conventional: 2.9 *p* = 0.87	NR	NR	mMPTA, mean (follow‐up NR) Computer‐assisted: 89.8 Conventional: 89.8 *p* = 0.92
Shetty, 2014 [72]	1155 Obese: 520 Nonobese: 635	HKA, 180° neutral convention, mean (48 mo.) <30: 179.7 ± 1.7 [172.0–185.5] ≥30: 179.6 ± 1.8 [173.1–185.0] >40: 179.4 ± 1.7 [175.0–182.6]	NR	NR	NR
Yoo, 2018 [79]	371 Obese: 78 Nonobese: 293	NR	TCA, mean (83.1 mo.) <25: 0.2 ± 1.2 25–30: 0.6 ± 1.5 ≥30: 0.6 ± 1.5 *p* = 0.12	FTA, mean (83.1 mo.) <25: 6.1 ± 1.8 25–30: 5.6 ± 1.6 ≥30: 5.2 ± 1.8 *p* = 0.006	NR

Post‐operative HKA angle using a 180° neutral convention was reported in 733 obese knees across three studies [43, 46, 72]. Across these studies, mean post‐operative alignment in obese strata was typically close to neutral, with small between‐group differences. Other studies reported HKA using alternative conventions (e.g., degrees varus/valgus around 0°, or medians/IQRs) [3, 30, 43, 50, 51, 69].

Regarding component alignment targets, Yoo et al. reported both continuous alignment measures and ‘within target’ proportions [79]. Post‐operative tibial component alignment angle did not differ significantly across BMI groups (overall *p* = 0.12), and the proportion within the specified tibial alignment target was similar across groups (BMI ≥ 30 93.4%, *p* = 0.49). Likewise, the proportion within the femorotibial alignment target did not differ (BMI ≥ 30 90.2%, *p* = 0.95), despite a statistically significant difference in the continuous femorotibial angle across BMI strata (overall *p* = 0.006) [79].

The mechanical medial proximal tibial angle (mMPTA) was reported post‐operatively in 310 obese knees across four studies [14, 30, 46, 69], demonstrating an average value of 89.2° (SD = 2.5°) across the two studies that provided mean values and the associated SD [14, 30].

Where direct obese versus non‐obese comparisons were performed, between‐group differences were small (generally <1°) and most frequently not statistically significant (*p* values commonly >0.05) [43, 46, 72, 79]. Even in studies reporting statistically significant differences in continuous alignment measures (e.g., femorotibial angle), the magnitude of deviation remained within accepted alignment targets and did not translate into differences in the proportion of cases achieving predefined radiographic goals [79]. Similarly, comparative analyses did not demonstrate clinically meaningful differences in mMPTA between obese and non‐obese patients [14, 30, 46, 69].

## DISCUSSION

The primary findings of this review were that mechanically aligned TKA in obese patients (BMI ≥ 30 kg/m^2^) is associated with favourable clinical, radiographic and survivorship outcomes. Mechanically aligned TKA was consistently associated with substantial post‐operative improvements in PROMs that frequently approximated or exceeded established PASS thresholds at the study level across multiple instruments (e.g., KSS, WOMAC and FJS‐12). However, as PASS is a patient‐level construct and included studies primarily reported post‐operative means rather than responder proportions, these comparisons provide clinical context regarding symptom states rather than direct evidence of PASS attainment. Despite modest decrements compared with non‐obese cohorts in some studies, post‐operative PROM performance in obese populations remained favourable overall. Importantly, the majority of comparative studies demonstrated no statistically significant differences in PROMs between obese and non‐obese patients. Although survivorship outcomes were heterogeneous in definition and reporting, reported cumulative revision incidence was low, and revision‐free survivorship was generally favourable across included mechanically aligned cohorts, commonly exceeding 95% at shorter‐term follow‐up and remaining above 90% in several longer‐term series. Similarly, radiographic outcomes demonstrated that MA reliably achieved near‐neutral limb alignment in obese patients, with mean post‐operative HKA angles typically clustering close to neutral and between‐group differences generally <1° and rarely statistically significant. Complication and revision rates were comparable to those reported in broader TKA populations. Importantly, these findings primarily reflect outcomes after mechanically aligned TKA and should not be interpreted as evidence that alignment philosophy independently influences outcomes in obese patients, as comparative evidence for alternative alignment strategies remains extremely limited.

Evidence evaluating KA, FA or other AA philosophies in obese patients undergoing TKA remains sparse. Only one included study investigated a kinematic implant using unspecified alignment in obese patients and reported comparable short‐term PROMs across BMI strata without an apparent penalty in early outcomes, and another included study investigated FA in obese patients and reported comparable short‐term PROMs across BMI strata. While these findings preliminarily suggest that TKA using FA may be feasible in selected obese patients, they do not permit meaningful comparison with mechanically aligned TKA or establish whether alignment philosophy influences outcomes in obesity. More broadly, although FA has demonstrated promising functional outcomes in mixed‐BMI populations, the absence of robust obese‐specific comparative data, particularly regarding survivorship, complications, and radiographic durability, precludes conclusions regarding the relative merits of different alignment strategies in this population. Accordingly, the present review supports acceptable outcomes following mechanically aligned TKA in obese patients but does not establish whether the alignment strategy itself matters in obese TKA.

These findings align with existing literature evaluating obesity as a risk factor in mechanically aligned TKA [31, 76, 81]. Numerous studies have demonstrated that obese patients experience substantial improvements in pain and function following TKA, albeit with slightly lower absolute PROMs compared with non‐obese patients, particularly at longer follow‐up intervals [7, 13, 29, 76]. Several contemporary studies have demonstrated that obese patients may experience greater pre‐ to post‐operative improvements in PROMs than non‐obese patients, likely reflecting larger gains from a higher baseline symptom burden [11, 52]. The present review reinforces this pattern, as several included studies reported statistically significant but clinically modest differences in WOMAC or KSS function scores favouring non‐obese patients at longer follow‐up, while overall post‐operative scores in obese cohorts remained high and consistent with good clinical outcomes [14, 47, 51, 56, 79]. This suggests that obesity primarily influences baseline symptom severity rather than the ability to achieve meaningful post‐operative improvement, regardless of potential important differences in baseline characteristics from different countries [11, 13, 17, 27, 52, 70].

Importantly, PROM findings should be interpreted within the context of the available evidence, which was predominantly derived from mechanically aligned TKA cohorts, with only limited evaluation of alternative alignment philosophies in obese patients. Consequently, comparisons across BMI strata primarily provide insight into the consistency of outcomes of TKA using MA philosophy, while evidence evaluating alternative alignment approaches in obese patients remains limited. As only one included study investigated a kinematic implant with unspecified alignment in obese patients and one study investigated FA in this population, direct comparison of patient‐reported outcomes across alignment strategies was not feasible. While mechanically aligned TKA demonstrated favourable PROMs that frequently met acceptable symptom thresholds across BMI strata, the limited comparative evidence precludes the determination of whether alignment philosophy meaningfully influences functional outcomes in obese populations. Future comparative studies evaluating mechanical, kinematic and other patient‐specific alignment strategies across stratified obesity classes are needed to determine whether alignment choice should be tailored to obesity severity.

Survivorship outcomes in this review were similarly consistent with existing evidence demonstrating acceptable implant longevity in obese patients undergoing mechanically aligned TKA [13, 32]. Although obesity has historically been associated with higher mechanical loading, accelerated polyethylene wear, and increased risk of aseptic loosening, the included studies reported low cumulative revision incidence and favourable revision‐free survivorship, with short‐term aseptic revision incidence of 0.5%–1.3% and mid‐ to long‐term revision‐free survivorship values exceeding 88.9% where reported [1, 39]. Differences in survivorship between obese and non‐obese patients were small or non‐significant in comparative analyses, suggesting that improvements in fixation methods, perioperative management, and advances in surgical techniques may mitigate some of the biomechanical risks traditionally attributed to obesity [5, 18, 67]. Importantly, the interpretation of revision indications in obese patients warrants caution. While aseptic loosening and infection were the most frequently reported causes of revision across included obese cohorts, these patterns are not necessarily unique to obesity and are also commonly reported in broader primary TKA populations. Given the limited number of studies directly comparing revision indications between obese and non‐obese patients, the present review cannot determine whether obesity meaningfully alters the distribution of revision mechanisms or simply influences the magnitude of otherwise typical failure patterns. This distinction is important, as obesity may increase overall mechanical and infectious risk without fundamentally changing the predominant etiologies of revision observed after contemporary TKA.

Radiographic findings further support the durability of MA in obese patients. Across studies using a 180° neutral HKA convention, post‐operative alignment clustered tightly around neutral, with minimal differences between BMI strata. These results support the concept that MA remains a reliable strategy for restoring the mechanical axis even in patients with increased soft‐tissue bulk and limb deformity [2, 46, 81]. Achieving accurate bone cuts can be technically challenging in obese patients, particularly during tibial preparation, where increased soft‐tissue envelope thickness and limited visualization can complicate guide positioning. However, several studies described strategies aimed at mitigating these technical challenges; for example, computer‐assisted navigation and accelerometer‐based guidance systems demonstrated comparable HKA alignment and tibial component positioning between obese and non‐obese cohorts, suggesting that technology‐assisted techniques may help maintain alignment accuracy in more technically demanding cases [30, 43, 72]. Similarly, PSI has been proposed as an adjunct to improve cutting accuracy by reducing reliance on external anatomical landmarks that may be obscured by soft tissue [14].

Of note, most included studies stratified patients by BMI, a global measure of body habitus that does not account for the regional distribution of adipose tissue around the knee joint. None of the radiographic alignment studies directly measured or adjusted for local periarticular adiposity when assessing for alignment accuracy. Emerging evidence suggests that local soft‐tissue adiposity, such as periarticular or prepatellar fat thickness, may be more clinically relevant than BMI for certain post‐operative outcomes, particularly surgical site infection. Imaging‐based measures of local adiposity have been shown to correlate more closely with complication risk than BMI alone [33]. Consequently, while the present findings suggest that obesity defined by BMI does not compromise the ability to achieve neutral MA, BMI may not accurately reflect the degree of localized adiposity around the knee; this distinction may partially explain why obese patients, as defined by BMI, can still achieve alignment targets at rates comparable to non‐obese patients.

In addition to global limb alignment, several studies reported component‐level alignment metrics that indirectly reflect sagittal and coronal plane accuracy. Post‐operative tibial component alignment angles and mechanical medial proximal tibial angles were typically centred around 89–90°, with minimal differences between BMI strata and no consistent increase in alignment outliers among obese patients [14, 30, 46, 69]. Although posterior tibial slope was not consistently reported, the available component alignment data suggest that sagittal plane tibial positioning can be reproduced with acceptable accuracy in obese patients when standard MA techniques or navigation‐assisted methods are employed.

Evidence for KA, FA or other AA philosophies in obese patients undergoing TKA remains limited. The single included study evaluating a kinematic implant using unspecified alignment strategy in TKA reported comparable post‐operative PROMs between obese and non‐obese patients at a minimum 2‐year follow‐up [45]. These findings align with broader KA literature in mixed‐BMI populations, suggesting potential improvements in early function and patient satisfaction [19, 25, 49, 66]. However, the absence of long‐term survivorship, complication and radiographic data in obese cohorts undergoing KA or FA precludes meaningful comparison with MA and prevents definitive conclusions regarding durability and safety in this higher‐risk population. Importantly, the absence of long‐term data should not be interpreted as evidence of inferiority, but rather as an absence of adequately powered longitudinal evaluation. The limited investigation of alternative alignment strategies likely reflects the historical emphasis on MA in higher‐risk populations, driven by concerns regarding implant durability, load distribution, and the potential for accelerated wear in the setting of increased body mass [8, 58, 71, 78].

Consequently, while KA or FA may provide comparable short‐term functional outcomes, their role in obese populations remains incompletely defined [12, 66]. The lack of high‐quality comparative evidence highlights the need for prospective, adequately powered studies and registry‐based analyses specifically evaluating alignment philosophy in obese and morbidly obese patients with long‐term follow‐up and standardized reporting of clinical, radiographic and survivorship outcomes.

This review is subject to several limitations. Although three RCTs were included, the majority of studies were retrospective, introducing potential selection bias and residual confounding for covariates such as baseline deformity severity, comorbidities, activity level and surgeon experience. Methodological quality was assessed using the MINORS criteria and the ROB2 tool to contextualize these limitations. Further, substantial heterogeneity existed in outcome reporting, including variability in PROM instruments, follow‐up duration, survivorship definitions and complication reporting, which limited the feasibility of formal meta‐analysis. Accordingly, outcomes were synthesized descriptively and pooled only when reporting conventions were sufficiently comparable. Additionally, obesity was frequently analyzed using binary or inconsistent BMI thresholds (≥ 30, ≥35 and ≥40 kg/m^2^), potentially obscuring differences between obesity classes. Where possible, stratified subgroup data were extracted to better characterize gradients in outcomes across BMI categories. Radiographic outcomes were also reported using differing conventions and summary statistics, precluding pooled quantitative analysis; as such, radiographic findings were organized by measurement convention and reported explicitly to allow cross‐study interpretation despite the absence of meta‐analysis. Finally, evidence for alternative alignment philosophies in obese patients was extremely limited, restricting the ability to draw comparative conclusions. This limitation was particularly pronounced given the marked imbalance in the available evidence base, with 14,662 obese knees undergoing mechanically aligned TKA compared with only a single study evaluating KA and another study evaluating FA in obese patients. As a result, despite the review being framed around alignment philosophy, the findings largely reflect outcomes of mechanically aligned TKA rather than comparative performance across alignment strategies. Accordingly, conclusions regarding kinematic, functional or other patient‐specific alignment approaches should be interpreted as hypothesis‐generating rather than comparative evidence. This highlights the need for prospective, adequately powered, long‐term comparative studies to determine whether alignment strategy should be tailored to obesity severity in contemporary TKA practice.

## CONCLUSION

Evidence evaluating the influence of alignment strategy on outcomes in obese patients undergoing TKA remains insufficient to determine whether alignment philosophy meaningfully affects clinical, radiographic or survivorship outcomes, or establish whether alignment choice should be tailored to obesity severity. Although mechanically aligned TKA demonstrated generally acceptable post‐operative outcomes in obese cohorts, the available literature was overwhelmingly dominated by mechanically aligned studies, with only limited evaluation of alternative approaches, such as KA or FA.

## AUTHOR CONTRIBUTIONS

All authors contributed to study design, data collection, analysis and manuscript preparation.

## FUNDING INFORMATION

The authors have no funding to report.

## CONFLICT OF INTEREST STATEMENT

Dr. Darren de SA has the following disclosures, none of which are related to this publication. He is a board member of the Heron Therapeutics Advisory Board and has served as a consultant for L.E.K. Consulting, Atheneum Partners and Stryker. Additionally, he has participated in the Speakers Bureau for ConMed Linvatec and is a member of the Pendopharm Regional Working Group. Dr. Amit Meena reports consulting roles with DePuy Mitek Sports Medicine and Maxx Orthopedics, none of which are affiliated with this publication. The other authors declare no conflicts of interest.

## ETHICS STATEMENT

The authors have nothing to report.

## Supporting information


**Appendix S1:** Electronic Database Search Strategy (Ovid MEDLINE, Embase, Emcare) for Studies Reporting Outcomes of Total Knee Arthroplasty in Obese Patients Using Various Alignment Philosophies.

## Data Availability

The data that support the findings of this study are available in the Supporting Information of this article.

## References

[1] Abdel MP , Bonadurer GF , Jennings MT , Hanssen AD . Increased aseptic tibial failures in patients with a BMI ≥35 and well‐aligned total knee arthroplasties. J Arthroplasty. 2015;30(12):2181–2184. 26220103 10.1016/j.arth.2015.06.057

[2] Andrews S , Weldon E , Stickley C , Nakasone C . Post total knee arthroplasty alignment of 347 consecutive obese patients receiving a fixed distal femoral cut of 6° valgus. J Orthop. 2020;18:113–116. 32021015 10.1016/j.jor.2019.10.006 PMC6994727

[3] Anwar R , Kini SG , Sait S , Bruce WJM . Early clinical and radiological results of total knee arthroplasty using patient‐specific guides in obese patients. Arch Orthop Trauma Surg. 2016;136(2):265–270. 26742495 10.1007/s00402-015-2399-z

[4] Armstrong JG , Morris TR , Sebro R , Israelite CL , Kamath AF . Prospective study of central versus peripheral obesity in total knee arthroplasty. Knee Surg Relat Res. 2018;30(4):319–325. 30466252 10.5792/ksrr.18.025 PMC6254871

[5] Baker P , Petheram T , Jameson S , Reed M , Gregg P , Deehan D . The association between body mass index and the outcomes of total knee arthroplasty. J Bone Jt Surg. 2012;94(16):1501–1508. 10.2106/JBJS.K.01180 22992819

[6] Bansal MR , Back D , Earnshaw P , Sandiford NA . Tibial alignment technique and its influence on clinical and functional outcomes following total knee arthroplasty. J Clin Orthop Trauma. 2020;11:534–538. 10.1016/j.jcot.2020.04.023 PMC7394794 32774025

[7] Baum G , Jacobs H , Lazovic D , Maus U , Hoffmann F , Seeber GH . The influence of obesity on functional outcomes and patient satisfaction 8 weeks after total knee arthroplasty: results of the prospective FInGK study. BMC Musculoskelet Disord. 2022;23(1):949. 36324114 10.1186/s12891-022-05874-w PMC9630069

[8] Bellemans J , Colyn W , Vandenneucker H , Victor J . The Chitranjan Ranawat award: is neutral mechanical alignment normal for all patients? The concept of constitutional varus. Clin Orthop Relat Res. 2012;470(1):45–53. 21656315 10.1007/s11999-011-1936-5 PMC3237976

[9] Bhattacharjee SK , Kundu Choudhury A , Priyadarshi S , Prasad A , Ahlawat A . Functional outcome in obese patients undergoing image‐based cruciate retaining robotic‐assisted total knee arthroplasty using the subvastus approach: a short‐term study. Cureus. 2024;16(9):e68430. 39360092 10.7759/cureus.68430 PMC11445690

[10] Bonnefoy‐Mazure A , Martz P , Armand S , Sagawa Y , Suva D , Turcot K , et al. Influence of body mass index on sagittal knee range of motion and gait speed recovery 1‐year after total knee arthroplasty. J Arthroplasty. 2017;32(8):2404–2410. 28545773 10.1016/j.arth.2017.03.008

[11] Bosler AC , Deckard ER , Buller LT , Meneghini RM . Obesity is associated with greater improvement in patient‐reported outcomes following primary total knee arthroplasty. J Arthroplasty. 2023;38(12):2484–2491. 37595768 10.1016/j.arth.2023.08.031

[12] Boutros M , Awad G , Mouawad A , Mansour E . Kinematic versus mechanically aligned total knee arthroplasty: a meta‐analysis of randomized controlled trials. J Knee Surg. 2026;39(6):283–295. 41285388 10.1055/a-2741-1246

[13] Boyce L , Prasad A , Barrett M , Dawson‐Bowling S , Millington S , Hanna SA , et al. The outcomes of total knee arthroplasty in morbidly obese patients: a systematic review of the literature. Arch Orthop Trauma Surg. 2019;139(4):553–560. 30778723 10.1007/s00402-019-03127-5 PMC6420900

[14] Braun S , Allen J , Somerville L , Howard JL , Lanting BA , Vasarhelyi EM . Patient‐specific instrumentation versus standard of care in total knee arthroplasty in an obese population: minimum 5‐year follow‐up. J Arthroplasty. 2026;41(2):458–464. 40562084 10.1016/j.arth.2025.06.060

[15] Chalidis BE , Petsatodis G , Christodoulou AG , Christoforidis J , Papadopoulos PP , Pournaras J . Is obesity a contraindication for minimal invasive total knee replacement? A prospective randomized control trial. Obes Surg. 2010;20(12):1633–1641. 19756888 10.1007/s11695-009-9968-6

[16] Conner‐Spady BL , Marshall DA , Bohm E , Dunbar MJ , Loucks L , Noseworthy TW . Patient acceptable symptom state (PASS): thresholds for the EQ‐5D‐5L and Oxford hip and knee scores for patients with total hip and knee replacement. Qual Life Res. 2023;32(2):519–530. 36367656 10.1007/s11136-022-03287-9

[17] Cushnaghan J , Bennett J , Reading I , Croft P , Byng P , Cox K , et al. Long‐term outcome following total knee arthroplasty: a controlled longitudinal study. Ann Rheum Dis. 2009;68(5):642–647. 18664545 10.1136/ard.2008.093229

[18] Deshmukh RG , Hayes JH , Pinder IM . Does body weight influence outcome after total knee arthroplasty? A 1‐year analysis. J Arthroplasty. 2002;17(3):315–319. 11938508 10.1054/arth.2002.30776

[19] Dossett HG , Estrada NA , Swartz GJ , LeFevre GW , Kwasman BG . A randomised controlled trial of kinematically and mechanically aligned total knee replacements: two‐year clinical results. The Bone & Joint Journal. 2014;96–B(7):907–913. 10.1302/0301-620X.96B7.32812 24986944

[20] Eckhoff DG , Bach JM , Spitzer VM , Reinig KD , Bagur MM , Baldini TH , et al. Three‐dimensional mechanics, kinematics, and morphology of the knee viewed in virtual reality. J Bone Joint Surg Am. 2005;87(Suppl 2):71–80. 16326726 10.2106/JBJS.E.00440

[21] El Kayali MKD , Pichler L , Gwinner C , Folkerts T . Local soft tissue thickness is a superior predictor of accuracy of implant alignment compared to body mass index in total knee arthroplasty. Arthroplasty. 2025;7(1):59. 41327505 10.1186/s42836-025-00347-6 PMC12670727

[22] Elcock KL , MacDonald DJ , Clement ND , Scott CEH . Total knee arthroplasty in patients with severe obesity: outcomes of standard keeled tibial components versus stemmed universal base plates. Knee Surg Relat Res. 2023;35(1):9. 37041576 10.1186/s43019-023-00184-4 PMC10088243

[23] Elmajee M , Mersal M , Ben Nafa W , Elsayed A , Alsonbaty M , Sherif IA , et al. Exploring knee alignment: demystifying traditional and emerging approaches. Cureus. 2025;17(9):e91423. 41040791 10.7759/cureus.91423 PMC12487995

[24] Estes CS , Schmidt KJ , McLemore R , Spangehl MJ , Clarke HD . Effect of body mass index on limb alignment after total knee arthroplasty. J Arthroplasty. 2013;28(8 Suppl):101–105. 23890833 10.1016/j.arth.2013.02.038

[25] Ettinger M , Tuecking LR , Savov P , Windhagen H . Higher satisfaction and function scores in restricted kinematic alignment versus mechanical alignment with medial pivot design total knee arthroplasty: a prospective randomised controlled trial. Knee Surg Sports Traumatol Arthrosc. 2024;32(5):1275–1286. 38501253 10.1002/ksa.12143

[26] Evans JT , Walker RW , Evans JP , Blom AW , Sayers A , Whitehouse MR . How long does a knee replacement last? A systematic review and meta‐analysis of case series and national registry reports with more than 15 years of follow‐up. Lancet. 2019;393(10172):655–663. 30782341 10.1016/S0140-6736(18)32531-5 PMC6381229

[27] Franklin PD , Miozzari H , Christofilopoulos P , Hoffmeyer P , Ayers DC , Lübbeke A . Important patient characteristics differ prior to total knee arthroplasty and total hip arthroplasty between Switzerland and the United States. BMC Musculoskelet Disord. 2017;18(1):14. 28077124 10.1186/s12891-016-1372-5 PMC5225636

[28] Ge L , Wang J , Fang H , Wang Y , Shen Z , Cai G . Effects of total knee arthroplasty on symptoms, function and activity over 5 years in knee osteoarthritis: a propensity‐score matched study. J Exp Orthop. 2025;12(1):e70185. 40123679 10.1002/jeo2.70185 PMC11928881

[29] Giesinger JM , Loth FL , MacDonald DJ , Giesinger K , Patton JT , Simpson AHRW , et al. Patient‐reported outcome metrics following total knee arthroplasty are influenced differently by patients' body mass index. Knee Surg Sports Traumatol Arthrosc. 2018;26(11):3257–3264. 29417168 10.1007/s00167-018-4853-2 PMC6208940

[30] Goto K , Hirota J , Miyamoto Y , Katsuragawa Y . The accuracy of a portable accelerometer‐based navigation system for tibial alignment can be reliable during total knee arthroplasty for obese patients. J Knee Surg. 2024;37(4):303–309. 37192656 10.1055/a-2094-8822

[31] Gunst S , Fessy MH . The effect of obesity on mechanical failure after total knee arthroplasty. Ann Transl Med. 2015;3(20):310. 26697470 10.3978/j.issn.2305-5839.2015.10.37 PMC4669315

[32] Hamoui N , Kantor S , Vince K , Crookes P . Long‐term outcome of total knee replacement: does obesity matter? Obes Surg. 2006;16(1):35–38. 16417755 10.1381/096089206775222140

[33] Heifner JJ , Sakalian PA , Rowland RJ , Corces A . Local adiposity may be a more reliable predictor for infection than body mass index following total knee arthroplasty: a systematic review. J Exp Orthop. 2023;10(1):110. 37930482 10.1186/s40634-023-00680-2 PMC10628095

[34] Higgins JPT , Altman DG , Gotzsche PC , Juni P , Moher D , Oxman AD , et al. The Cochrane Collaboration's tool for assessing risk of bias in randomised trials. BMJ. 2011;343(2):d5928. 22008217 10.1136/bmj.d5928 PMC3196245

[35] Hiranaka T , Suda Y , Saitoh A , Tanaka A , Arimoto A , Koide M , et al. Current concept of kinematic alignment total knee arthroplasty and its derivatives. Bone Jt Open. 2022;3(5):390–397. 35532356 10.1302/2633-1462.35.BJO-2022-0021.R2 PMC9134837

[36] Howell SM , Howell SJ , Kuznik KT , Cohen J , Hull ML . Does a kinematically aligned total knee arthroplasty restore function without failure regardless of alignment category? Clin Orthop Relat Res. 2013;471(3):1000–1007. 22996362 10.1007/s11999-012-2613-z PMC3563808

[37] Hsu RWW , Himeno S , Coventry MB , Chao EYS . Normal axial alignment of the lower extremity and load‐bearing distribution at the knee. Clin Orthop Relat Res. 1990;255:215–227. 2347155

[38] Ishimoto R , Mutsuzaki H , Shimizu Y , Yoshikawa K , Koseki K , Takeuchi R , et al. Association between obesity and short‐term patient‐reported outcomes following total knee arthroplasty: a retrospective cohort study in Japan. J Clin Med. 2024;13(5):1291. 38592115 10.3390/jcm13051291 PMC10932041

[39] Jackson MP , Sexton SA , Walter WL , Walter WK , Zicat BA . The impact of obesity on the mid‐term outcome of cementless total knee replacement. J Bone Joint Surg Br. 2009;91(8):1044–1048. 19651831 10.1302/0301-620X.91B8.22129

[40] Järvenpää J , Kettunen J , Kröger H , Miettinen H . Obesity may impair the early outcome of total knee arthroplasty. Scand J Surgery. 2010;99(1):45–49. 10.1177/145749691009900110 20501358

[41] Ji B , Guarin Perez SF , Pagnano MW , Sierra RJ , Perry KI . Alignment strategies in total knee arthroplasty and how to effectively achieve them. J Am Acad Orthop Surg. 2025;33(21):e1249–e1259. 40779766 10.5435/JAAOS-D-25-00123

[42] Kamat YD , Aurakzai KM , Adhikari AR . Total knee replacement in the obese patient: comparing computer assisted and conventional technique. ScientificWorldJournal. 2014;2014:272838. 24523634 10.1155/2014/272838 PMC3913015

[43] Kanna R , Brasanna A , Shetty GM , Ravichandran C . No influence of obesity on mid‐term clinical, functional, and radiological results after computer‐navigated total knee arthroplasty using a gap balancing technique. J Clin Orthop Trauma. 2021;16:136–142. 33717948 10.1016/j.jcot.2021.01.001 PMC7920157

[44] Karasavvidis T , Pagan Moldenhauer CA , Lustig S , Vigdorchik JM , Hirschmann MT . Definitions and consequences of current alignment techniques and phenotypes in total knee arthroplasty (TKA)—there is no winner yet. J Exp Orthop. 2023;10(1):120. 37991599 10.1186/s40634-023-00697-7 PMC10665290

[45] Kolin DA , Carroll KM , Ast MP , Mayman DJ , Haas SB , Cushner F . Do obese patients benefit from a kinematic, appropriately designed total knee prosthesis? J Orthop. 2022;34:147–151. 36060732 10.1016/j.jor.2022.07.023 PMC9437742

[46] Koutserimpas C , Gregori P , Andriollo L , Diquattro E , Servien E , Batailler C , et al. Impact of high body mass index on functionally aligned image‐based robotic total knee arthroplasty: Comparable functional outcomes but higher mechanical failures. J ISAKOS. 2025;12:100861. 40210164 10.1016/j.jisako.2025.100861

[47] Krushell RJ , Fingeroth RJ . Primary total knee arthroplasty in morbidly obese patients. J Arthroplasty. 2007;22(6 Suppl 2):77–80. 17823021 10.1016/j.arth.2007.03.024

[48] Kuklinski D , Marques CJ , Bohlen K , Westphal KC , Lampe F , Geissler A . Thresholds for meaningful improvement in WOMAC scores need to be adjusted to patient characteristics after hip and knee replacement. J Orthop. 2022;29:50–59. 35125779 10.1016/j.jor.2022.01.002 PMC8803617

[49] Laende EK , Richardson CG , Dunbar MJ . A randomized controlled trial of tibial component migration with kinematic alignment using patient‐specific instrumentation versus mechanical alignment using computer‐assisted surgery in total knee arthroplasty. Bone Joint J. 2019;101–B(8):929–940. 10.1302/0301-620X.101B8.BJJ-2018-0755.R3 31362561

[50] Lai Y , Zhao W , Li X , Lv N , Zhou Z . Comparison of outcomes in obese patients after total knee arthroplasty with neutral or mild varus: a retrospective study with 8‐year follow‐up. Orthop Surg. 2024;16(5):1127–1133. 38556476 10.1111/os.14040 PMC11062869

[51] Lai YH , Cao J , Li ZX , Feng W , Xu H , Zhou ZK . Effect of body mass index on postoperative mechanical alignment and long‐term outcomes after total knee arthroplasty: a retrospective cohort study of 671 knees. Ann Transl Med. 2022;10(15):829. 36034999 10.21037/atm-22-3212 PMC9403940

[52] Lawrence KW , Sobba W , Rajahraman V , Schwarzkopf R , Rozell JC . Does body mass index influence improvement in patient reported outcomes following total knee arthroplasty? A retrospective analysis of 3918 cases. Knee Surg Relat Res. 2023;35(1):21. 37496075 10.1186/s43019-023-00195-1 PMC10373362

[53] Le GT , Van Duren BH , Ilo K , Berber R , Matar HE , Bloch BV . Cementless TKA use as an alternative to cemented TKA in high BMI patients: a systematic review and meta‐analysis. J Exp Orthop. 2024;11(3):e12067. 39011084 10.1002/jeo2.12067 PMC11247335

[54] Li D , Troelsen A , Ingelsrud L , Husted H , Gromov K . Females, younger patients and patients with high BMI have the highest pre‐operative knee awareness measured using the Forgotten Joint Score. Knee Surg Sports Traumatol Arthrosc. 2018;26(9):2587–2593. 28210786 10.1007/s00167-017-4446-5

[55] Liberati A , Altman DG , Tetzlaff J , Mulrow C , Gotzsche PC , Ioannidis JPA , et al. The PRISMA statement for reporting systematic reviews and meta‐analyses of studies that evaluate healthcare interventions: explanation and elaboration. BMJ. 2009;339:b2700. 19622552 10.1136/bmj.b2700 PMC2714672

[56] Lizaur‐Utrilla A , Miralles‐Muñoz FA , Sanz‐Reig J , Collados‐Maestre I . Cementless total knee arthroplasty in obese patients. J Arthroplasty. 2014;29(6):1192–1196. 24355257 10.1016/j.arth.2013.11.011

[57] Luan C , Xu DT , Chen NJ , Wang FF , Tian KS , Wei C , et al. How to choose kinematic or mechanical alignment individually according to preoperative characteristics of patients? BMC Musculoskelet Disord. 2020;21(1):443. 32635906 10.1186/s12891-020-03472-2 PMC7341594

[58] Matassi F , Pettinari F , Frasconà F , Innocenti M , Civinini R . Coronal alignment in total knee arthroplasty: a review. J Orthop Traumatol. 2023;24(1):24. 37217767 10.1186/s10195-023-00702-w PMC10203068

[59] McCormick BP , Trent S , Geng X , Lee JW , Boucher HR . Robotic‐assisted technology does not influence functional outcomes among obese and morbidly obese total knee arthroplasty patients. J Exp Orthop. 2023;10(1):76. 37523073 10.1186/s40634-023-00634-8 PMC10390435

[60] Meissner N , Ramos‐Pascual S , Ortwig K , van Rooij F , Schrednitzki D , Stoeve J , et al. Outcomes of total hip arthroplasty in obese patients with and without preoperative weight loss: a systematic review and meta‐analysis. J Exp Orthop. 2026;13(1):e70651. 41574211 10.1002/jeo2.70651 PMC12821894

[61] Mont MA , Mathur SK , Krackow KA , Loewy JW , Hungerford DS . Cementless total knee arthroplasty in obese patients. J Arthroplasty. 1996;11(2):153–156. 8648308 10.1016/s0883-5403(05)80009-9

[62] Nisar S , Palan J , Rivière C , Emerton M , Pandit H . Kinematic alignment in total knee arthroplasty. EFORT Open Rev. 2020;5(7):380–390. 32818065 10.1302/2058-5241.5.200010 PMC7407864

[63] Nishitani K , Yamamoto Y , Hamada D , Ito H , Nakamura S , Kuriyama S , et al. Patient acceptable symptom state for New Knee Society Score after primary total knee arthroplasty. J Orthop Sci. 2026;31(3):609–613. 41152048 10.1016/j.jos.2025.10.004

[64] Ojard C , Habashy A , Meyer M , Chimento G , Ochsner JL . Effect of obesity on component alignment in total knee arthroplasty. Ochsner J. 2018;18(3):226–229. 30275786 10.31486/toj.18.0005 PMC6162131

[65] Puah KL , Yeo W , Tan MH . Clinical and radiographic outcomes of computer‐navigated total knee arthroplasty are not adversely affected by body mass index. J Orthop. 2020;19:54–58. 32021037 10.1016/j.jor.2019.11.002 PMC6994793

[66] Rahman WA , Muqri KY , Suhail HM , Shajri MM , Omar YZ , Alahmari MA , et al. Functional outcomes and patient satisfaction in kinematic vs mechanical alignment total knee arthroplasty: a systematic review. Cureus. 2025;17(10):e93939. 41209940 10.7759/cureus.93939 PMC12590092

[67] Rhind JH , Baker C , Roberts PJ . Total hip arthroplasty in the obese patient: tips and tricks and review of the literature. Indian J Orthop. 2020;54(6):776–783. 33133400 10.1007/s43465-020-00164-w PMC7572957

[68] Ritter MA , Davis KE , Meding JB , Pierson JL , Berend ME , Malinzak RA . The effect of alignment and BMI on failure of total knee replacement. J Bone Jt Surg. 2011;93(17):1588–1596. 10.2106/JBJS.J.00772 21915573

[69] Rivkin G , Kandel L , Perets I , Tsohar T , Nasrawy T , Liebergall M . Total knee arthroplasty using a computerized assisted stereotaxic navigation system with bluetooth communication in obese patients—a randomized controlled study. Comput Assist Surg. 2023;28(1):2162970. 10.1080/24699322.2022.2162970 36637291

[70] Runhaar J , Koes BW , Clockaerts S , Bierma‐Zeinstra SMA . A systematic review on changed biomechanics of lower extremities in obese individuals: a possible role in development of osteoarthritis. Obes Rev. 2011;12(12):1071–1082. 21812903 10.1111/j.1467-789X.2011.00916.x

[71] Segura‐Nuez J , Martín‐Hernández C , Segura‐Nuez JC , Segura‐Mata JC . Methods of alignment in total knee arthroplasty, systematic review. Orthop Rev. 2024;16:117769. 10.52965/001c.117769 PMC11142931 38827414

[72] Shetty GM , Mullaji AB , Bhayde S , Lingaraju AP . No effect of obesity on limb and component alignment after computer‐assisted total knee arthroplasty. Knee. 2014;21(4):862–865. 24799079 10.1016/j.knee.2014.04.004

[73] Singh V , Fiedler B , Huang S , Oh C , Karia RJ , Schwarzkopf R . Patient Acceptable Symptom State for the Forgotten Joint Score in primary total knee arthroplasty. J Arthroplasty. 2022;37(8):1557–1561. 35346809 10.1016/j.arth.2022.03.069

[74] Slim K , Nini E , Forestier D , Kwiatkowski F , Panis Y , Chipponi J . Methodological index for non‐randomized studies (minors): development and validation of a new instrument. ANZ J Surg. 2003;73(9):712–716. 12956787 10.1046/j.1445-2197.2003.02748.x

[75] Teimouri M , Salehi A , Shahsavan M , Rezaei H , Dayani Dardashti A . Effectiveness of total knee arthroplasty on pain reduction and functional improvement in elderly patients: a quasi‐experimental study. Adv Biomed Res. 2025;14:86. 40958919 10.4103/abr.abr_409_24 PMC12435714

[76] van Tilburg J , Rathsach Andersen M . Mid‐ to long‐term complications and outcome for morbidly obese patients after total knee arthroplasty: a systematic review and meta‐analysis. EFORT Open Rev. 2022;7(5):295–304. 35510746 10.1530/EOR-21-0090 PMC9142821

[77] Varacallo MA , Luo TD , Mabrouk A , Johanson NA . Total knee arthroplasty techniques. StatPearls. Treasure Island, FL: StatPearls Publishing; 2025. 29763071

[78] Yang HY , Seon JK , Ayob KA . Evolving philosophies of alignment in TKA: from mechanical uniformity to personalised harmony. Medicina (Kaunas). 2026;62(2):307. 41752706 10.3390/medicina62020307 PMC12941868

[79] Yoo JH , Oh HC , Park SH , Kim JK , Kim SH . Does obesity affect clinical and radiological outcomes in minimally invasive total knee arthroplasty? Minimum 5‐year follow‐up of minimally invasive TKA in obese patients. Clin Orthop Surg. 2018;10(3):315–321. 30174807 10.4055/cios.2018.10.3.315 PMC6107814

[80] Young SW , Zeng N , Tay ML , Fulker D , Esposito C , Carter M , et al. A prospective randomised controlled trial of mechanical axis with soft tissue release balancing vs functional alignment with bony resection balancing in total knee replacement‐a study using Stryker Mako robotic arm‐assisted technology. Trials. 2022;23(1):580. 35858944 10.1186/s13063-022-06494-4 PMC9296895

[81] Zurrón Lobato M , Bartolomé García S , Aragonés Maza P , Valverde Villar A , Ramírez Feito C , González González MS , et al. The use of navigation during total knee replacement improves precision in achieving mechanical alignment in obese patients: a short‐term multicenter randomized clinical trial. JB JS Open Access. 2025;10(4):e25.00220. 10.2106/JBJS.OA.25.00220 PMC12533999 41112698

