# Supplementary material for: Mechanically aligned total knee arthroplasty demonstrates acceptable outcomes in obese patients, with insufficient evidence for alternative alignment strategies: A systematic review
Source: J Exp Orthop. 2026 Jul 30;13(3):e70860. doi: 10.1002/jeo2.70860 (PMC13420840; doi:10.1002/jeo2.70860)
Supplement: Supplementary file 1 — Appendix S1: Electronic Database Search Strategy (Ovid MEDLINE, Embase, Emcare) for Studies Reporting Outcomes of Total Knee Arthroplasty in Obese Patients Using Various Alignment Philosophies. [file JEO2-13-e70860-s001.docx]

**APPENDIX**

**Appendix 1.** Electronic Database Search Strategy (Ovid MEDLINE, Embase, Emcare) for Studies Reporting Outcomes of Total Knee Arthroplasty in Obese Patients Using Various Alignment Philosophies

| **Database: Ovid MEDLINE(R) ALL <1946 to September 22, 2025>**  **Search Strategy:**  1. Obesity/ 236968  2. Overweight/ 37725  3. Obes*.mp 503179  4. Body Mass Index/ 162677  **5. 1 OR 2 OR 3 OR 4**  **585937**  6. Kinematic.mp 29680  7. Mechanical.mp 2779  8. Alignment.mp 208311  **9. 6 OR 7 OR 8**  **239523**  10. Arthroplasty, Replacement, Knee/ 36425  11. Knee Prosthesis/ 14899  12. TKA.mp 20684  13. Knee*.mp 235646  **14. 10 OR 11 OR 12 OR 13 236010**  **15. 5 AND 9 AND 14 347** |
| --- |
| **Database: Embase <1974 to 2025 September 22>**  **Search Strategy:**  1. Obesity/ 642137  2. Overweight/ 642137  3. Obes*.mp 871610  4. Body Mass Index/ 782959  **5. 1 OR 2 OR 3 OR 4**  **1392386**  6. Kinematic.mp 34362  7. Mechanical.mp 4654  8. Alignment.mp 202346  **9. 6 OR 7 OR 8**  **239728**  10. Arthroplasty, Replacement, Knee/ 9651  11. Knee Prosthesis/ 12932  12. TKA.mp 24326  13. Knee*.mp 339270  **14. 10 OR 11 OR 12 OR 13 339778**  **15. 5 AND 9 AND 14 2002** |
| **Database: Ovid Emcare <1995 to 2025 Week 38>**  **Search Strategy:**  1. Obesity/ 194447  2. Overweight/ 103627  3. Obes*.mp 254764  4. Body Mass Index/ 260682  **5. 1 OR 2 OR 3 OR 4**  **423311**  6. Kinematic.mp 15822  7. Mechanical.mp 803  8. Alignment.mp 44157  **9. 6 OR 7 OR 8**  **59950**  10. Arthroplasty, Replacement, Knee/ 1766  11. Knee Prosthesis/ 5047  12. TKA.mp 13317  13. Knee*.mp 149763  **14. 10 OR 11 OR 12 OR 13 149918**  **15. 5 AND 9 AND 14 1064** |
